# Physical activity and mental health in children and adolescents with intellectual disabilities: a meta-analysis using the RE-AIM framework

**DOI:** 10.1186/s12966-022-01312-1

**Published:** 2022-07-07

**Authors:** Wen Yang, Xiao Liang, Cindy Hui-Ping Sit

**Affiliations:** Department of Sports Science and Physical Education, The Chinese University of Hong Kong, Shatin, N.T., Hong Kong

**Keywords:** Adolescent, Children, Physical activity, Cognitive function, Psychological health, Intellectual disability

## Abstract

**Background:**

Children and adolescents with intellectual disabilities (IDs) tend to have lower levels of physical activity and poorer mental health than their typically developing peers. Studies on the effects of physical activity on the mental health of children with IDs using the Reach, Effectiveness, Adoption, Implementation, and Maintenance (RE-AIM) framework are scarce.

**Methods:**

A systematic literature review using six databases (CINAHL, Eric, PsycINFO, PubMed, SPORTDiscus, and Web of Science) was conducted from January 2000 to September 2021. Studies reporting at least one physical activity intervention and mental health outcome in children and adolescents with IDs aged between 5 and 17 years were included in the meta-analysis. Preferred Reporting Items for Systematic Review and Meta-Analysis guideline, Comprehensive Meta-Analysis, and the RE-AIM framework were utilized.

**Results:**

A total of 15 studies that met the inclusion criteria were included in the meta-analysis. The effects of physical activity on mental health in children and adolescents with IDs were significant and large (Hedges’ *g* = 0.897, *p* < 0.01), with medium effects on psychological health (Hedges’ *g* = 0.542, *p* < 0.01) and large effects on cognitive function (Hedges’ *g* = 1.236, *p* < 0.01). Randomized controlled trial (RCT) design and intervention components (> 120 minutes per week, therapeutic, and aerobic exercise) demonstrated the strongest effects. Moreover, study background (publication year, study location, and sample size), participant characteristics (age and sex), and Maintenance (RE-AIM framework) moderated the effects of physical activity on mental health. Based on the RE-AIM framework, there were higher proportions in the dimensions of Reach and Effectiveness than Adoption, Implementation, and Maintenance.

**Conclusions:**

Physical activity appears to have positive effects on mental health, including psychological health and cognitive function, in children and adolescents with IDs. Physical activity interventions using the RE-AIM framework are recommended to assess short- and long-term impacts and translate scientific evidence into practice.

**Trial registration:**

The protocol for this meta-analysis was registered with PROSPERO (CRD42021256543).

**Supplementary Information:**

The online version contains supplementary material available at 10.1186/s12966-022-01312-1.

## Background

Intellectual disabilities (IDs) refer to a broad range of mental impairments preventing individuals from participating in daily life to the same text as typically developing (TD) individuals [1]. The classification of ID level is determined using the fifth edition of the Diagnostic and Statistical Manual of Mental Disorders (DSM-V) [2]. Globally, the prevalence of IDs is approximately 1%, with a higher ratio in men than women, with roughly 85% falling within the mild level [3]. Compared to peers with TD, the rate of mental health problems in children and adolescents with IDs is three to four times higher [4, 5], and they are more likely to be exposed to socio-economic disadvantages [6].

According to the World Health Organization (WHO), mental health could be determined based on psychological factors, and mental health promotion involves the improvement of psychological health [7]. Psychological health has been used to represent positive and negative feelings in personal and social life, including psychological well- and ill-being [8]. Mental health includes basic cognitive skills and the ability to cope with function in social roles [9]. Psychological health and cognitive function are important elements of mental health and have gained attention in physical activity studies [10–12].

Recent WHO guidelines on physical activity and sedentary behavior recommended that children and adolescents living with a disability should engage in at least 60 minutes per day of moderate-to-vigorous physical activity across the week, with at least three days of vigorous-intensity aerobic activities per week [13]. Physical activity has been found to have beneficial effects on mental health in children and adolescents with disabilities [14]. However, children and adolescents with IDs engage in lower levels of physical activity than their peers with TD [15–17].

Previous reviews with meta-analyses indicated that physical activity had positive moderate-to-large effects on psychosocial well-being (Hedges’ *g* = 0.682, *p* < 0.01) and positive influence on emotional problems in children and adolescents with IDs [18, 19]. These reviews explored the moderating effects of study background (e.g., sample size and study design), participant characteristics (e.g., age and ID level), and intervention components (e.g., type and setting); however, cognitive function outcomes were under-researched. Moreover, some reviews and meta-analyses reported the effects of physical activity on cognitive function in children and adolescents with other disabilities. For instance, physical activity had positive effects on cognitive function in children and adolescents with social, emotional, and behavioral disabilities [20]. Exercise interventions had positive small-to-moderate effects (Hedges’ *g* = 0.342, *p* < 0.01) on executive function in children and adolescents with autism spectrum disorder [21]. Exercise had a positive moderate-to-large effect (Hedges’ *g* = 0.611, *p* < 0.01) on cognitive function in children and adolescents with attention-deficit/hyperactive disorder [22]. Participant characteristics, such as age, and intervention components, such as session and content, moderated the effects of physical activity on cognitive function in children with disabilities [22, 23]. Extant studies called for an examination of the moderating effects of sex and intervention duration [20].

Previous reviews indicated the moderating effects of publication year on mental health outcomes in TD populations [24, 25], and social, cultural, and environmental factors were potential barriers to physical activity participation for children with disabilities [26]. Due to the intellectual impairments in children and adolescents with IDs, their mental health outcomes were self-reported and reported by their teachers and parents simultaneously [27]. Teachers and parents could identify more severe mental health problems than children themselves [28]. However, the moderating roles of publication year, study location, and outcome reporter in the effects of physical activity on mental health were under-explored in children and adolescents with IDs.

Antikainen and Ellis (2011) [29] and McGoey et al. (2015) [30] indicated that the interventions had no long-term follow-up assessments and focused on internal rather than external validity, which may reduce the generalizability to ecological settings [29, 30]. The five-step Reach, Effectiveness, Adoption, Implementation, and Maintenance (RE-AIM) framework [31, 32] has been used to guide physical activity interventions and evaluate external validity of theory-based physical activity interventions [29, 33, 34], and to examine the impact of translational research in the disability research [35].

Several research gaps existed in previous literature. First, while previous systematic reviews with meta-analyses examined the effects of physical activity on psychological health in children and adolescents with IDs [18, 19], the effects of physical activity on mental health, including psychological health and cognitive function, in children and adolescents with IDs were under-researched. Second, the quality of previous systematic reviews and meta-analyses in children and adolescents with IDs was evaluated using risk of bias, which showed moderate to high risk [18, 19]; however, external validity, such as generalizability or applicability, based on the RE-AIM framework in children and adolescents with IDs was not examined. Third, while previous systematic reviews and meta-analyses in children and adolescents with IDs reported the moderating roles of study background, participant characteristics, and intervention components using subgroup analyses [18, 19], other moderating effects, such as study location, overcome reporter, and the RE-AIM framework, were under-explored. Therefore, our meta-analysis aimed to (1) determine the effects of physical activity on mental health, including psychological health and cognitive function, in children and adolescents with IDs, (2) evaluate physical activity interventions in children and adolescents with IDs using the RE-AIM framework, and (3) examine the moderating roles of the study background, participant characteristics, intervention components, and the RE-AIM framework.

## Methods

### Protocol

This meta-analysis was conducted in accordance with the Preferred Reporting Items for Systematic Review and Meta-Analysis guidelines (PRISMA) [36]. The PRISMA guidelines contain 27 items (see Additional file 1), and a protocol for this meta-analysis was registered with PROSPERO (CRD42021256543).

### Search strategy

A systematic search was conducted using CINAHL (EBSCO), Education Resources Information Center (ERIC, EBSCO), PsycINFO (OVID), Pubmed (NIBI), SPORTDiscus (EBSCO), and Web of Science databases on September 10, 2021. The publication date was from January 1, 2000 to September 9, 2021, and the search was conducted by two researchers (WY and XL). Four terms were used in the search: (1) intellectual disability, (2) children and adolescents, (3) physical activity, and (4) mental health. The search strategy for the SPORTDiscus database is presented in Additional file 2.

### Selection procedure and eligibility criteria

**Fig. 1:**
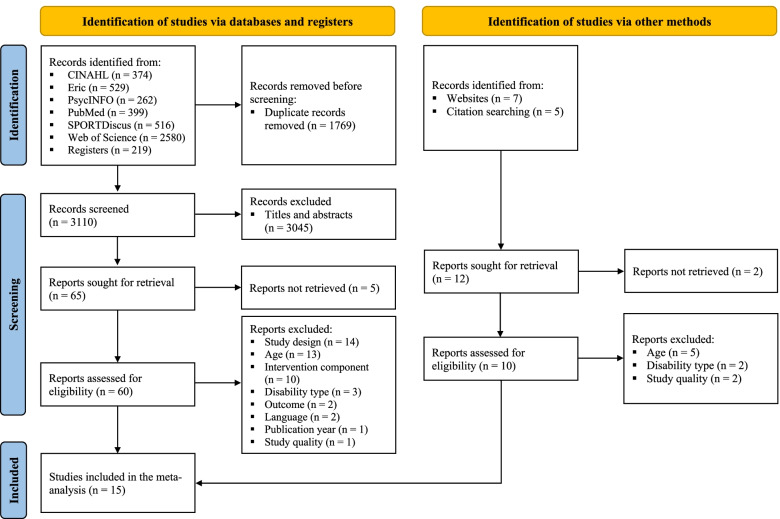
The PRISMA flow diagram of the search and screening process

After all duplicates were removed, two researchers (WY and XL) independently screened the titles, abstracts, and full text of the search studies. References were imported and uploaded to Endnote X9. The researchers resolved any disagreements through discussion, and a third researcher (CHPS) deliberated to reach a final decision. The Kappa statistic was calculated (fair [0.40–0.59], good [0.60–0.74], and excellent [≥0.75]) to evaluate inter-rater reliability [22]. A total of 4879 original articles were obtained from six databases. After 1769 duplicates were removed, 3110 articles remained; however, 3045 articles were excluded after their titles and abstracts were screened, five articles were excluded as reports could not be retrieved, and 60 abstracts met the inclusion criteria with an inter-rater reliability of k = 0.62. A further 46 articles were removed after their full texts were screened for the following reasons: 14 due to study design, 13 due to age, 10 due to intervention components, three due to disability type, two due to outcome, two due to language, and one each due to publication year and study quality. One article was included via citation searching. Therefore, 15 articles were included, with an inter-rater reliability of k = 0.73. Figure 1 shows the PRISMA flow diagram of the search and screening process.

The inclusion and exclusion criteria followed the PICOS framework, which included population, intervention, comparison, outcome, and study design [37]. Inclusion criteria for eligible studies were as follows: (1) population of children and adolescents aged between 5 and 17 years with IDs, including mental retardation, mental disabilities, intellectual impairments, and cognitive impairments, (2) intervention being physical activity, sports, exercise, games, and training, (3) comparison of physical inactivity, sedentary behavior, and daily activity, (4) at least one measured and reported outcome on mental health, and (5) study design being intervention, including randomized controlled trial (RCT) and non-RCT, published in a peer-reviewed journal with full-text in English from January 2000 to September 2021.

The exclusion criteria were (1) children and adolescents with developmental disabilities or learning disabilities, (2) lack of comparison groups, (3) observational studies, qualitative studies, or reviews, publication as a conference poster, conference abstract, protocol, or commentary, and publication earlier than January 2000.

### Data extraction

Data extraction followed the Cochrane handbook for systematic reviews of interventions [38]. Two researchers extracted data from each of the eligible studies: (1) study background, including name of the first author, publication year, study location, outcome reporter (self-report and teacher/parent proxy-report), study design (RCT and non-RCT), and sample size, (2) participant characteristics, including age, sex, and ID level, (3) intervention components, including type, setting, and protocol (duration per week, total session, and total duration), (4) measures and outcomes of mental health, including psychological health (anxiety, depression, emotional self-control, self-competence, self-esteem withdrawn, and self-worth) and cognitive function (accuracy, cognitive flexibility, executive function, inhibition control, reaction time, and working memory).

### Quality assessment

The 21-item validated quality evaluation tool based on the RE-AIM framework (see Additional file 3) was used to code eligible articles on the degree to which indicators of internal and external validity were reported [35], and improve the translatability and impact of health interventions [33]. In the RE-AIM framework, Reach refers to the percentage and risk characteristics of participants. A sample question was “What percent of potentially eligible participants were excluded and included, and how representative were they?” Effectiveness indicates the positive and negative consequences of the intervention. A sample question was “What are the positive and negative outcomes for participants, including quality of life?” Adoption is the proportion and representativeness of intervention personnel and protocol. A sample question was “What percent of settings and intervention agents within these settings were excluded and included, and how representative were they?” Implementation assesses the participants’ adherence to intervention, and the extent to which personnel deliver intervention as intended. A sample question was “To what extent were the various intervention components delivered as intended?” Maintenance evaluates the extent to which the intervention becomes a routine of daily life. A sample question was “What were the long-term effects, and indicators of program level maintenance?“ [32]. The quality assessment was conducted by two researchers (WY and XL) independently. The Kappa statistic values for consistency were 0.65 for Reach, 0.63 for Effectiveness, 0.72 for Adoption, 0.67 for Implementation, and 0.82 for Maintenance, indicating good to excellent inter-rater reliability. The disagreements were resolved by discussion, and researchers gained consensus in the coding by revisiting the included articles.

### Meta-analytic procedures

We used Comprehensive Meta-Analysis (CMA; version 3.0) to conduct the meta-analysis. Only studies that reported sufficient statistical data from pre- and post-test in experimental and control groups were included in the meta-analysis. A random-effects model was used to measure heterogeneity distributed effect size, as it uses sample error and between-study variance to estimate effect size [22]. Standardized mean differences (SMD) for continuous outcomes with different measurement units were calculated and weighted, and the mean (M), sample size (N), and standard deviation (SD) were the primary methods for effect size calculations. Hedges’ *g* and its 95% confidence interval (CI) were used in this meta-analysis, which could correct the overestimated effect size for small sample size, and Cohen’s *d* was used for large sample size [22, 39]. Effect size values of 0.20 indicates small, 0.50 indicates medium, and 0.80 indicates large effect size [40]. The statistical heterogeneity (*I*^*2*^) was assessed using a *p* value calculated for Q statistics, indicating small (≤25%), medium (50%), and large (≥75%) heterogeneity [21]. Except for the holistic meta-analyses for mental health, subgroup analyses of psychological health and cognitive function were conducted based on specific outcomes. The meta-regression was used to explore the moderating effects of study background (publication year, study location, outcome reporter, study design, and sample size), participant characteristics (age, sex, and ID level), intervention components (type, setting, duration per week, total session, and total duration), and RE-AIM framework.

The presence of outliers was investigated by analyzing relative residual values of included studies, and a standard score (z-score) more than or equal to a positive or negative value of 1.96 was considered a large residual value [41]. A sensitivity analysis (i.e., one study removed procedure in CMA software) was used to inspect the impact of retention/removal of outliers and their influence on the overall effect size. Studies should be retained when the overall effect size after removal remains significant and within the 95% CI [41]. To decrease potential publication bias, a funnel plot calculating the standard error and effect size was used, and Duval and Tweedie’s trim and fill method (i.e., random-effects model) was utilized to measure the publication-bias adjusted effect size and the number of studies required to balance the plot [22]. In addition, Begg and Mazumdar rank correlation and Egger’s regression intercept were performed to test for publication bias [42]. A statistical significance of *p* < 0.05 was set for all tests.

## Results

### Characteristics of included studies

Table 1 shows the descriptive characteristics of the 15 included studies. There were a total of 630 participants, with 313 participants involved in trials of psychological health, and the remaining 317 cognitive function. Of 15 included articles, nine [43–51] studies (60%) examined psychological health, and six [52–57] (40%) cognitive function. As for measures of mental health, six [43–48] studies (40%) focused on psychological well-being using the Checklist for Pupil Evaluation and Self-perception Profile, three [49–51] studies (20%) measured psychological ill-being using Child Behavior Checklist, 12-item Anxiety/Depression Scale, and Withdrawn Behavior Checklist, five [53–57] studies (33.3%) used reaction time measurements, such as Cognitive Performance, and two [52, 54] studies (13.3%) used Flanker test (inhibition control), Go/No-Go test (accuracy), NIH Toolbox Cognitive Battery (working memory), and Wisconsin Card Sorting Test-64 card version (executive function).

**Table 1:** Descriptive characteristics of included studies (n=15)

		Participants			Intervention				Mental health		
Author/Year/Location	Study design	N (% girl)	Age	ID level	Type	Setting	Content	Protocol (Total session/Duration per week/Total duration)	Dimension	Measure	Standardized mean difference
**Psychological health**											
Choi & Cheung (2016) [43]; Hong Kong	RCT	E:18 (33.3) C:12 (16.7)	7.39±0.5	Mild	Therapeutic exercise	School	Therapeutic recreation	24-session/180-min/1440-min	ESC	Checklist for Pupil Evaluation	ESC: 1.03^**^
Maïano et al. (2002) [44]; France	Non-RCT	E:8 (0) C:16 (0)	13.40±0.92 13.60±0.62	Mild	Competitive sports	Community	Basketball	56-session/120-min/6720-min	SW SC	Self-perception Profile	SW: 0.25 SC: 0.22
Maïano et al. (2001) [45]; France	RCT	E:16 (0) C:16 (0)	13.50±0.80	Mild to moderate	Competitive sports	Community	Basketball, running	30-session/120-min/720-min	SC	Self-perception Profile	SC: 0.28
Ninot & Maïano (2007) [46]; France	Non-RCT	E:32 (100) C:16 (100)	13-17	Mild to moderate	Competitive Sports	Community	Basketball, swimming	91-session/120-min/10920-min	SW SC	Self-perception Profile	SW: 0.50 SC: 0.37
Ninot et al. (2005) [47]; France	Non-RCT	E:32 (100) C:16 (100)	13-17	Mild to moderate	Competitive sports	Community	Swimming	139-session/120-min/16680-min	SW SC	Self-perception Profile	SW: 0.42 SC: 0.40
Ninot et al. (2000) [48]; France	Non-RCT	E:32 (100) C:16 (100)	13-17	Mild to moderate	Competitive sports	Community	Basketball, swimming	35-session/120-min/4200-min	SW SC	Self-perception Profile	SW: 0.73 SC: 0.70
Özer et al. (2012) [49]; Turkey	RCT	E:23 (0) C:15 (0)	14.50±1.20 14.50±0.80	Overall	Non-competitive sports	Community	Soccer	24-session/270-min/2160-min	SC A/D	Child Behaviour Checklist	SC-Parent-proxy:0.81^*^ SC-Teacher-proxy: 0.20 A/D-Parent-proxy: 0.12 A/D-Teacher-proxy: 0.62
Perić et al. (2021) [50]; Servia	RCT	E: 12 (0) C: 13 (0)	15.68±0.49 15.72±0.46	Mild to moderate	Competitive sports	Community	Soccer	32-session/120-min/1920-min	A/D	12-item Anxiety/Depression Scale	A/D-Parent-proxy: 1.315^**^
Ryuh et al. (2019) [51]; USA	Non-RCT	E:10 (0) C:10 (0)	10.80±0.60 10.40±0.70	Overall	Non-competitive sports	School	Soccer	20-session/450-min/1800-min	SEW	Withdrawn Behaviour Checklist	SEW: 1.45^**^
**Cognitive function**											
Chen et al. (2015) [52]; Taiwan	RCT	SOT: 46 (43.5) TTT: 45 (46.7) C: 41 (43.9)	10.90±3.90 10.60±3.60 10.70±4.00	Mild	Aerobic exercise, therapeutic exercise	Community	Table tennis, occupational therapy	48-session/180-min/2880-min	EF CF	EF: WCST-64 CF: Stroop color-word test	SOT-EF: 1.16^**^ SOT-CF: 1.97^**^ TTT-EF: 1.33^*^ TTT-CF: 1.90^**^
Giagazoglou et al. (2013) [53]; Greece	RCT	E:10 (0) C:9 (0)	15.30±2.10	Mild to moderate	Therapeutic exercise	School	Hippotherapy exercise	28-session/70-min/980-min	RT	Rise from the armchair	Auditory-CE: 1.54^**^ Auditory-OE: 2.07^**^ Visual: 1.33^**^
Mazzoli et al. (2021) [54]; Australia	RCT	E: 15 (33.3) C: 9 (44.4)	9.90±1.00	Mild to moderate	Cognitive exercise	School	Classroom break	50-session/50-min/250-min	ACC IC RT WM	IC: Flanker RT & ACC: GNGT WM: NIHTB-CB	ACC: 0.07 IC: 0.15 RT: 0.37 WM: 1.07^**^
Pise et al. (2018) [55]; India	RCT	E: 35 (31.4) C: 35 (48.6)	10-15	Mild to moderate	Cognitive exercise	NR	Yoga	60-session/300-min/3600-min	RT	Ruler test	RT: 3.13^**^
Vogt et al. (2013) [56]; Germany	Non-RCT	E:11 C:11 (46)	16.00±1.34	Overall	Aerobic exercise	School	Cycling	NR	RT	Cognitive performance	Visual-L: 0.33 Visual-P: 1.38^**^
Yildirim et al. (2010) [57]; Turkey	RCT	E:25 (24) C:25 (20)	14.52±1.50 14.80±1.29	Mild	Cognitive exercise	NR	Physical fitness	36-session/120-min/2160-min	RT	Newest Reaction Time Scale	Auditory: 0.95^**^ Visual: 0v86^**^

*ACC* accuracy, *A/D* anxiety & depression, *Auditory-CE* auditory reaction time with eyes closed, *Auditory-OE* auditory reaction time with eyes open, *C* control group, *CF* cognitive flexibility, *E* experimental group, *ESC* emotional self-control, *EF* executive function, *GNGT* Go/No-Go Task, *IC* inhibition control, *ID* intellectual disability, *NIHTB-CB* NIH Toolbox Cognitive Battery, *NR* not reported, *RCT* randomized controlled trial design, *RT* reaction time, *SC* self-competence, *SEW* self-esteem withdrawn, *SOT* standard occupational therapy, *SW* self-worth, *TTT* table tennis training, *Visual-L* visual reaction time by using lifting, *Visual-P* visual reaction time by using pressing, *WCST-64* Wisconsin Card Sorting Test-64 card version, *WM* working memory

^*^p<0.05; ^**^p<0.01

The sample size ranged from 20 to 132 participants with a mean age of 13.16 years, including five [43, 51, 52, 54, 55] studies (40%) with children (5–11 years) and 10 studies [44–50, 53, 56, 57] (60%) with adolescents (12–17 years) [8]. Regarding sex, six [44, 45, 49–51, 53] studies (40%) included all male participants, three [46–48] studies (20%) all female participants, and six [43, 52, 54–57] studies (40%) included men and women. Mental health outcomes in 12 studies [44–48, 51–57] (80%) were self-reported, and the other three studies [43, 49, 50] (20%) used reports provided by teachers or parents. Nine studies [43, 45, 49, 50, 52–55, 57] (60%) adopted the RCT design, and six [44, 46–48, 51, 56] studies (40%) employed non-RCT design. Ten studies [44–50, 53, 56, 57] (66.7%) were conducted in Europe (five in France, two in Turkey, and one each in Greece, Germany, and Servia), three [43, 52, 55] (20%) in Asia (one each in Hong Kong, Taiwan, and India), and one each [51, 54] (13.3%) in America and Australia. As for the identification of the ID, eight studies [44, 45, 47–50, 52, 53] (53.3%) used Wechsler Intelligence Scale for Children [58], two studies [46, 57] (13.3%) used American Association on Mental Retardation [59], two studies [51, 54] (13.3%) followed the DSM-V criteria [2], and one study [56] (6.7%) followed the American Association on Intellectual and Developmental Disabilities [60]. Four studies [43, 44, 52, 57] (26.7%) focused on mild ID, eight studies [45–48, 50, 53–55] (53.3%) focused on mild to moderate ID, and three studies [49, 51, 56] (20%) focused on overall ID.

Collectively, eight studies [44–50, 52] (53.3%) were conducted in community settings, such as Special Olympics, and five studies [43, 51, 53, 54, 56] (33.3%) were conducted in schools. There were five types of intervention: six studies [44–48, 50] (40%) used competitive sports, such as basketball, swimming, and soccer, three studies [54, 55, 57] (20%) used cognitive exercise, such as fitness training and yoga, three studies [43, 52, 53] (20%) used therapeutic exercise, two studies [49, 51] (13.3%) used non-competitive sports, such as unified sports soccer, and two studies [52, 56] (13.3%) used aerobic exercise, such as cycling. For intervention protocol, nine studies [43, 45, 48–53, 57] (60%) conducted interventions less than 50 sessions in total, and five studies [44, 46, 47, 54, 55] (33.3%) ≥50 intervention sessions in total. Furthermore, nine studies [44–48, 50, 53, 54, 57] (60%) included training of ≤120 minutes per week, and five studies [43, 49, 51, 52, 55] (33.3%) used training of > 120 minutes per week. The total intervention duration ranged from 250 minutes to 16,680 minutes.

### Quality assessment

Table 2 shows the proportion of physical activity interventions reporting components of the RE-AIM framework. The total score of the RE-AIM framework was 40.5%, and among the five dimensions, Reach showed the highest proportion (76%), followed by Effectiveness (56.7%), Implementation (37.8%), Adoption (23.3%), and Maintenance (8.9%). In the Reach dimension, all included studies reported methods of identifying the target population and inclusion criteria; however, only four studies [45, 49, 50, 52] (26.7%) reported the participation rate. In the Effectiveness dimension, measures/results for at least one follow-up got the highest proportion (100%); and only three studies [49–51] (20%) reported quality of life or potential negative outcomes. In the Adoption dimension, no studies reported adoption rate of delivery agent or setting, one study [49] (6.7%) reported inclusion and exclusion criteria of delivery agent or setting, and two studies [43, 56] (13.3%) reported level of expertise of delivery agent. In the Implementation dimension, all included studies reported intervention duration and frequency; no studies reported measures of cost of implementation. In the Maintenance dimension, two studies (13.3%) reported assessed outcome ≥6 months post intervention [44, 45] and indicators of program level maintenance [50, 54], and no studies included measures of cost of maintenance. Across all included studies, inclusion rates of individual RE-AIM evaluation ranged from 27.7 to 66%. The proportion of physical activity interventions reporting the RE-AIM framework of included studies is presented in Additional file 4.

**Table 2:** The proportion of physical activity interventions reporting components of the RE-AIM framework

RE-AIM framework	RE-AIM component	Proportion Reporting^**a**^, %
Reach	Method to identify target population	100%
	Inclusion criteria	100%
	Exclusion criteria	73.3%
	Participation rate	26.7%
	Representativeness	80%
	*Average Reach Dimension*	*76%*
Effectiveness	Measures/results for at least one follow-up	100%
	Intent to treat analysis method	60%
	Quality-of-life or potential negative outcomes	20%
	Percent attrition	46.7%
	*Average Effectiveness Dimension*	*56*.*7%*
Adoption	Description of intervention location	46.7%
	Description of staff who delivered intervention	46.7%
	Method to identify staff who delivered intervention (target delivery agent)	26.7%
	Level of expertise of delivery agent	13.3%
	Inclusion/exclusion criteria of delivery agent or setting	6.7%
	Adoption rate of delivery agent or setting	0%
	*Average Adoption Dimension*	*23*.*3%*
Implementation	Intervention duration and frequency	100%
	Extent protocol delivered as intended (%)	13.3%
	Measures of cost of implementation	0%
	*Average Implementation Dimension*	*37*.*8%*
Maintenance	Assessed outcomes ≥6 months post intervention	13.3%
	Indicators of program level maintenance	13.3%
	Measures of cost of maintenance	0%
	*Average Maintenance Dimension*	*8*.*9%*
*Total*		*40*.*5%*

^a^ Based on the denominator of 15 intervention trials

### Summary of findings

#### Effects of physical activity on mental health

**Fig. 2:**
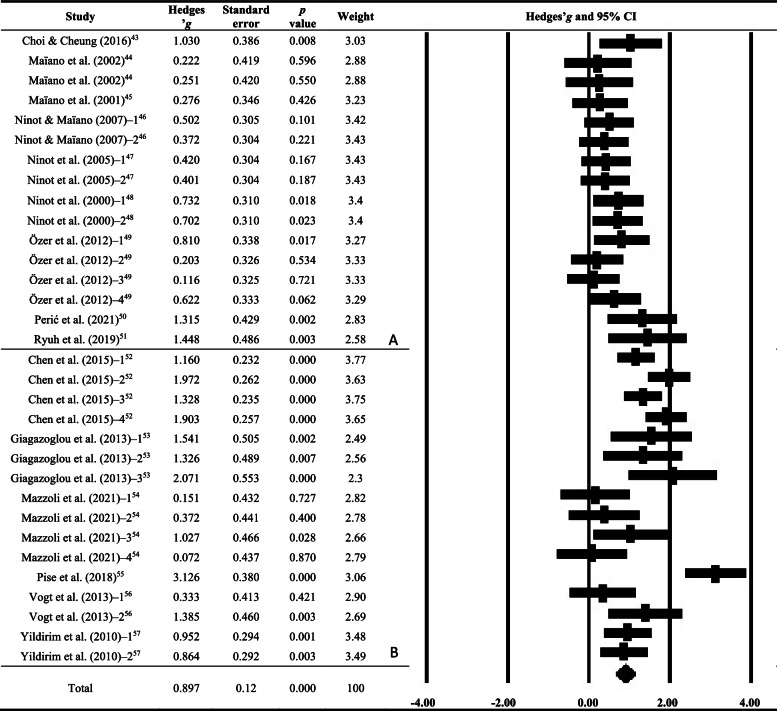
Forest plot for effects of physical activity (experimental) vs. control on measures of mental health in children and adolescents with intellectual disabilities. *Note.* A random-effects model was used to measure heterogeneity distributed effect size; A: psychological health; B: cognitive function; CI: confidence interval

Among 15 included studies, the pooled effect size for children and adolescents with IDs is shown in Fig. 2. There was a large effect of physical activity on overall mental health (Hedges’ *g* = 0.897, 95% CI = [0.659, 1.136], *p* < 0.01) with a medium heterogeneity (Q = 121.153, df = 31, *p* < 0.01, *I*^*2*^ = 74.413%). The effects of physical activity on psychological health from nine studies were medium (Hedges’ *g* = 0.542, 95% CI = [0.374, 0.709], *p* < 0.01) with no significant heterogeneity (Q = 14.804, df = 15, *p* > 0.05, *I*^*2*^ = 0%). The effect size on cognitive function from six studies was significant and large (Hedges’ *g* = 1.236, 95% CI = [0.871, 1.600], *p* < 0.01) with a large heterogeneity (Q = 66.683, df = 15, *p* < 0.01, *I*^*2*^ = 77.560%).

#### Moderator analyses

Table 3 shows the meta-regression results regarding the moderating effects of study background, participant characteristics, intervention components, and the RE-AIM framework. Publication year (Q = 5.41, df = 1, R^2^ = 22%, *p* < 0.05), study location (Q = 23.63, df = 2, R^2^ = 60%, *p* < 0.01), sample size (Q = 6.82, df = 1, R^2^ = 32%, *p* < 0.01), age (Q = 8.00, df = 1, R^2^ = 37%, *p* < 0.01), intervention type (Q = 14.14, df = 4, R^2^ = 39%, *p* < 0.01), intervention duration per week (Q = 5.83, df = 1, R^2^ = 25%, *p* < 0.05), and Maintenance (Q = 4.24, df = 1, R^2^ = 11%, *p* < 0.05) were significant moderators in the effects of physical activity on mental health in children and adolescents with IDs. Trends were observed in the moderating effects of study design (Q = 3.71, df = 1, R^2^ = 15%, *p* = 0.05) and sex (Q = 5.63, df = 2, R^2^ = 26%, *p* = 0.06). However, other potential factors had no moderating effects (*p* > 0.05).

**Table 3:** Moderators in the effects of physical activity on mental health in children and adolescents with intellectual disabilities

Variable	Level	Beta (95% CI)	Q	df	Adjusted ***R***^**2**^ (%)
Publication year	Range: 2000-2021	0.041 (0.007, 0.076)	5.41^*^	1	22
Study location (ref: Asia)	Europe	-1.038 (-1.472, -0.604)	23.63^**^	2	60
	America and Australia	-1.124 (-1.757, -0.492)			
Outcome provider (ref: Self-report)	Teacher/parent proxy-report	-0.293 (-0.895, 0.309)	0·91	1	2
Study design (ref: RCT)	Non-RCT	-0.465 (-0.938, 0.008)	3.71	1	15
Sample size	Range: 20-145 participants	0.013 (0.003, 0.022)	6.82^**^	1	32
Age (ref: Children 5-11 years)	Adolescents (12-17 years)	-0.617 (-1.045, -0.187)	8.00^**^	1	37
Sex (ref: Boys-only)	Girls-only	-0.252 (-0.848, 0.344)	5.63	2	26
	Mix-sex	0.395 (-0.093, 0.882)			
ID level (ref: Mild ID)	Mild to moderate ID	-0.271 (-0.797, 0.255)	2.16	2	13
	Overall ID	-0.467 (-1.108, 0.174)			
Intervention type (ref: Therapeutic exercise)	Aerobic exercise	-0.255 (-0.989, 0.479)	14.14^**^	4	39
	Cognitive exercise	-0.548 (-1.202, 0.106)			
	Non-competitive sport	-1.005 (-1.602, -0.408)			
	Competitive sport	-0.931 (-1.630, -0.232)			
Intervention setting (ref: School)	Community	-0.183 (-0.718, 0.351)	4.02	2	1
	NR	0.642 (-0.229, 1.512)			
Intervention duration per week (ref: >120 min/week)	≤120 min/week	-0.566 (-1.028, -0.105)	5.83^*^	1	25
	NR	-0.409 (-1.412, 0.594)			
Total intervention session	Range: 20-139 sessions	-0.004 (-0.012, 0.004)	1.07	1	0
Total intervention duration	Range: 250-16680 min	-0.000 (-0.000, 0.000)	3.45	1	16
Reach	Range: 40%-100%	-0.510 (-2.284, 1.264)	0.32	1	0
Effectiveness	Range: 25%-100%	0.503 (-0.401, 1.408)	1.19	1	4
Adoption	Range: 0%-83.3%	0.361 (-0.410, 1.133)	0.84	1	3
Implementation	Range: 33.3%-66.7%	0.419 (-1.988, 2.825)	0.12	1	0
Maintenance	Range: 0%-33.3%	-1.410 (-3.400. -0.083)	4.24^*^	1	11
Overall RE-AIM	Range: 27.7%-66%	0.397 (-1.576, 2.370)	0.16	1	1

*ID* intellectual disability, *NR* not reported, *RCT* randomized controlled trial, *ref* reference group

^*^p<0·05; ^**^p<0·01

#### Subgroup analyses

Subgroup analyses (see Additional file 5) demonstrated that physical activity had positive effects on mental health in children (Hedges’ *g* = 1.272, *p* < 0.01), adolescents (Hedges’ *g* = 0.656, *p* < 0.01), boy-only (Hedges’ *g* = 0.762, *p* < 0.01), girl-only (Hedges’ *g* = 0.519, *p* < 0.01), and mixed groups (Hedges’ *g* = 1.153 *p* < 0.01). Moreover, significant and positive effects of physical activity on mental health were found in children and adolescents with mild ID (Hedges’ *g* = 1.137, *p* < 0.01), mild to moderate ID (Hedges’ *g* = 0.865, *p* < 0.01), and overall ID (Hedges’ *g* = 0.630, *p* < 0.01). Furthermore, intervention studies in various locations, such as Asia (Hedges’ *g* = 1.730, *p* < 0.01), Europe (Hedges’ *g* = 0.656, *p* < 0.01), and America and Australia (Hedges’ *g* = 0.586, *p* < 0.05), found positive effects on mental health in children and adolescents with IDs. Outcomes reported by children and adolescents with IDs (Hedges’ *g* = 0.953, *p* < 0.01) and their parents and teachers (Hedges’ *g* = 0.634, *p* < 0.01) showed positive physical activity effects on mental health. Interventions using RCT design (Hedges’ *g* = 1.056, *p* < 0.01) had stronger effects on mental health than those with non-RCT design (Hedges’ *g* = 0.560, *p* < 0.01).

Therapeutic (Hedges’ *g* = 1.521, *p* < 0.01) and aerobic exercise (Hedges’ *g* = 1.251, *p* < 0.01) showed stronger effects on mental health than cognitive exercise (Hedges’ *g* = 0.950, *p* < 0.05), non-competitive sports (Hedges’ *g* = 0.568, *p* < 0.01), and competitive sports (Hedges’ *g* = 0.509, *p* < 0.01). Children and adolescents with IDs who exercised more than 120 minutes per week (Hedges’ *g* = 1.244, *p* < 0.01) had better mental health outcomes than those who exercised ≤120 minutes per week (Hedges’ *g* = 0.654, *p* < 0.01). Intervention settings, such as school (Hedges’ *g* = 0.932, *p* < 0.01) and community (Hedges’ *g* = 0.764, *p* < 0.01), had significant and positive effects on mental health.

### Sensitivity analysis and publication bias

**Fig. 3:**
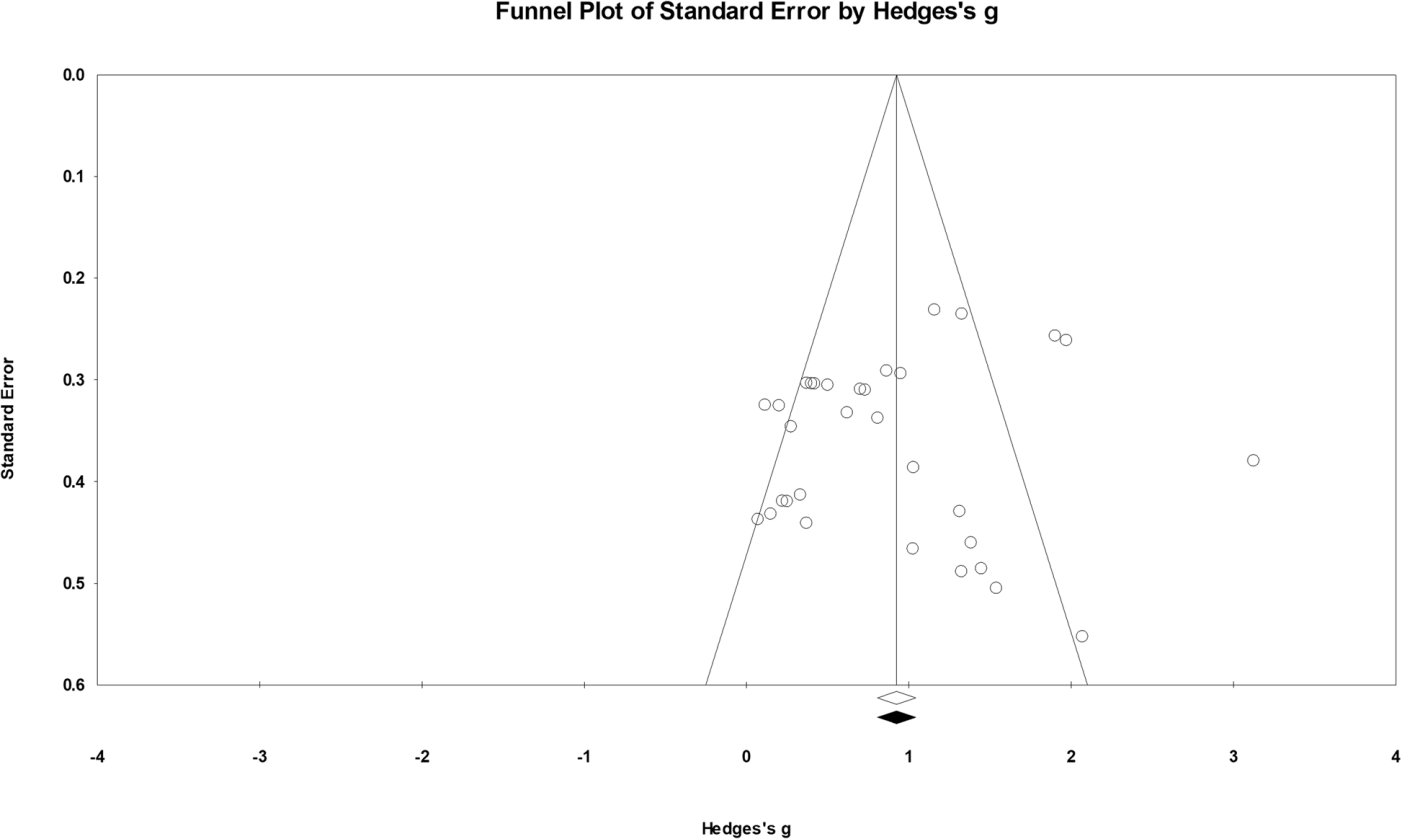
Funnel plot for visual inspection of publication bias

Eight studies [43, 48, 50–53, 55, 57] were found to be outliers (z-score ranged from 2.269 to 8.233), and “one study removed” sensitivity analysis was performed. Results showed that all pooled outcomes were stable to the sequential removal of outliers (Hedges’ *g* ranged from 0.827 to 0.924), which remained significant and within the 95% CI. Therefore, these eight studies were retained in the final analysis. The funnel plot of publication bias is illustrated in Fig. 3. Based on Duval and Tweedie’s trim and fill method, publication-bias adjusted effect size was 0.924 (95% CI [0.806, 1.042]), and no studies were needed to balance the plot. The results of Begg and Mazumdar’s (z = 0.438, *p* > 0.05) and Egger’s tests (intercept = − 0.987, 95% CI [− 4.189, 2.215], *p* > 0.05) indicated that the conclusion of the meta-analysis was not susceptible to publication bias.

## Discussion

Our meta-analysis demonstrated that physical activity had significant and large effects on mental health in children and adolescents with IDs, with medium effects on psychological health and large effects on cognitive function. Similarly, previous reviews found medium effects of physical activity on psychosocial health in children with IDs [19] and working memory in children and adolescents with learning disabilities [23]. Possible mechanisms of the effects of physical activity on mental health may be explained by physiological reasons, such as physical activity benefitting mental function [61], modulating neuroinflammation, inhibiting neuronal integrity, and enhancing neurotrophin levels, neurogenesis, and vascularization [62]. A review noted that physical activity might induce change in neurological, psychological, and behavioral parameters, such as increased neural activity within the prefrontal cortex and the functional activity of monoamines related to mood, and improved coping efficacy through the mediation of hippocampal neurogenesis activity and activation of the hypothalamic-pituitary-adrenal axis [8].

In our meta-analysis, intervention components (> 120 minutes per week, therapeutic, and aerobic exercise) were moderators in the effects of physical activity on mental health in children and adolescents with IDs, which was consistent with previous reviews. For instance, high intervention frequency was more effective than low intervention frequency, and combined exercise interventions were more effective than non-aerobic exercise on cognitive function in persons with IDs [23]. Intervention type, such as resistance training, was found to have large effects on mental health in children and adolescents with IDs [19]. Therapeutic exercise has received some attention for mental health problems in children and adolescents with IDs; however, evidence is insufficient and inconclusive [63]. Although intervention setting had no moderating effects, interventions conducted in schools and communities had positive effects on mental health in children and adolescents with IDs in this meta-analysis. Previous studies indicated that intervention setting was a moderator in physical activity intervention [18]. School settings had significant effects on physical activity in children with IDs [64]. School environments were found to affect physical activity levels in children with IDs, and they were more active at schools where support for physical activity was available [65]. Therefore, more school-based intervention studies are required to improve the mental health of children and adolescents with IDs.

Our meta-analysis also indicated that study background, such as publication year and sample size, were significant moderators, and RCT design had larger effects compared with non-RCT design, which were consistent with the findings in TD populations [24, 25]. Intervention studies with more rigorous designs on physical activity and its effects on mental health in children and adolescents with IDs are required. Moreover, study location had significant moderating effects, and it reflected different cultures and social values, which could be potential barriers to physical activity participation for children with disabilities [26]. Outcome reporter did not have any moderating effects; however, a previous study found that teacher or parent reporters could identify more severe mental health problems [28]. Multiple informants, such as clinicians, teachers, and parents, are important to assess the actual effects of physical activity on mental health.

Using the RE-AIM framework, we found that the dimensions of Reach and Effectiveness had higher proportions than Adoption, Implementation, and Maintenance, which was consistent with previous reviews using the RE-AIM framework in physical activity interventions in TD populations [30, 33, 66]. We also found that Maintenance was a significant moderator in the effects of physical activity on mental health, with only two studies assessing long-term follow-up. Previous studies also recommended long-term and sustainable physical activity participation and scalable interventions for children and adolescents with disabilities [67]. To improve the quality of physical activity interventions in children and adolescents with IDs, particularly in Adoption, Implementation, and Maintenance, the following approaches are suggested: (1) reporting the inclusion/exclusion criteria and adoption rate of delivery agent/setting, (2) calculating costs of equipment and personnel, (3) adding follow-up assessments that occur at least 6 months after the completion of physical activity interventions, and (4) reporting the degree of sustaining the physical activity interventions [66].

In our meta-analysis, ID level had no moderating effects, whereas previous studies showed that ID level moderated physical activity outcomes [19] and was a strong predictor in participants who achieved WHO physical activity guidelines [68]. A previous study revealed that children with a more severe ID level were more sedentary and had less physical activity participation than those with a lower ID level [69]. The reason for the discrepancy may be that this study only included participants with mild ID, mild to moderate ID, and overall ID. Moreover, age and sex had moderating effects, which were consistent with previous studies [28]. Future studies should recruit more participants with severe or profound ID, and consider the mental age and sex distribution of children and adolescents with IDs.

To the best of our knowledge, this was the first meta-analysis using the RE-AIM framework that examined the effects of physical activity on mental health in children and adolescents with IDs. Some limitations must be addressed. First, there was a lack of evidence on the dimensions of Adoption, Implementation, and Maintenance. These three dimensions should be identified in future research to enhance the quality of physical activity interventions. Although all included studies reported at least one follow-up, only two studies assessed the outcomes ≥6 months post intervention, which suggested the need for more robust strategies to keep participants engaged in physical activity intervention and improve the extent to which intervention becomes a routine of daily life. Second, no studies focused on severe or profound ID, which may limit the generalizability of the study. Third, most included studies had small sample size, highlighting the urgent need for future physical activity intervention studies to include more participants and those with severe or profound ID. Fourth, the social value connected with physical activity may vary between cultures, future research should consider the racial or cultural characteristics of physical activity and mental health. Fifth, physical activity interventions included diverse contents, such as basketball, swimming, soccer, fitness training, yoga, and cycling, but the recommended intervention contents and participants’ feedback were under-investigated. Future studies should collect feedback from participants and target effective and preferred contents of physical activity interventions in children and adolescents with IDs.

## Conclusions

Our meta-analysis indicated that physical activity appears to have positive effects on mental health, including psychological health and cognitive function, in children and adolescents with IDs. RCT design and intervention components (> 120 minutes per week, therapeutic, and aerobic exercise), showed the strongest effects. Study background (publication year, study location, and sample size), participant characteristics (age and sex), and Maintenance (RE-AIM framework) moderated the effects of physical activity on mental health in children and adolescents with IDs. Long-term follow-up and degree of sustaining the intervention are needed in future studies.

## Supplementary Information

**Additional file 1.** PRISMA 2020 Checklist.

**Additional file 2.** Search strategy in SPORTDiscus database.

**Additional file 3.** Coding sheet for publications reporting on the RE-AIM framework components.

**Additional file 4. **Proportion of PA interventions reporting the RE-AIM framework of included studies (*n* = 15).

**Additional file 5.** Forest plot of subgroup analyses.

## Data Availability

Data sharing is not applicable to this article as no datasets were generated or analyzed during the current study.

## Abbreviations

CI: Confidence interval
CMA: Comprehensive Meta-Analysis
DSM-V: The fifth edition of the Diagnostic and Statistical Manual of Mental Disorders
ID: Intellectual disability
PICOS: Population, intervention, comparison, outcome, and study design
PRISMA: Preferred Reporting Items for Systematic Review and Meta-Analysis guidelines
RCT: Randomized controlled trial
RE-AIM: Reach, Effectiveness, Adoption, Implementation, and Maintenance
SMD: Standardized mean difference
TD: Typically developing
WHO: World Health Organization
