# Supplementary material for: Physical activity and mental health in children and adolescents with intellectual disabilities: a meta-analysis using the RE-AIM framework
Source: Int J Behav Nutr Phys Act. 2022 Jul 7;19:80. doi: 10.1186/s12966-022-01312-1 (PMC9261031; doi:10.1186/s12966-022-01312-1)
Supplement: Supplementary file 2 — Additional file 2. Search strategy in SPORTDiscus database. [file 12966_2022_1312_MOESM2_ESM.docx]

**Additional file 2. Search strategy in SPORTDiscus database**

| **Term** | **Search Strategy** |
| --- | --- |
| Intellectual disability | AB intellectual disability OR AB mental retardation OR AB mental disabled OR AB mentally disabled OR AB mental development OR AB intellectual development OR AB cognitive impairment OR AB intellectual impairment OR AB mental disability OR AB mental disabilities |
| Children and adolescents | AB children OR AB child OR AB adolescent* OR AB adolescence OR AB teenager* OR AB youth OR AB school*aged OR AB student* OR AB school OR AB 5-17 years OR AB school-aged |
| Physical activity | AB physical activit* OR AB physical fitness OR AB physical exercise OR AB physical education OR AB leisure activit* OR AB motor activit* OR AB sport* participation OR AB fitness OR AB cardiovascular fitness OR AB exercis* OR AB acute exercise OR AB chronic exercise OR AB healthy exercise OR AB yoga OR AB pilates OR AB aerobic exercise OR AB aerobic OR AB aerobic training OR AB resistance exercise OR AB anaerobic exercise OR AB intervention OR AB baseball OR AB football OR AB soccer OR AB basketball OR AB racket sports OR AB racquet sports OR AB badminton OR AB tennis OR AB cricket OR AB track and field OR AB running OR AB sprint OR AB long distance running OR AB jump OR AB bike OR AB bicycling OR AB boxing OR AB taekwondo OR AB judo OR AB golf OR AB gymnastics OR AB hockey OR AB climbing OR AB hiking OR AB Tai Ji OR AB martial arts OR AB swimming OR AB aquatic exercise OR AB diving OR AB skating OR AB snow sports OR AB skiing OR AB sports for persons with disabilities OR AB youth sports OR AB wrestling OR AB weight Lifting OR AB game* OR AB virtual sports game OR AB recreation* OR AB motor skills OR AB motor intervention OR AB functional exercise OR AB motor learning OR AB behaviour skills training OR AB task specific training OR AB strength training OR AB horse riding OR AB hippotherapy OR AB trampoline OR AB snowshoeing OR AB skating OR AB exergaming OR AB skateboarding OR AB dance OR AB walking OR AB treadmill |
| Mental health | AB mental health OR AB mental wellbeing OR AB mental well-being OR AB mental wellness OR AB psychological wellbeing OR AB psychological well-being OR AB well-being OR AB wellbeing OR AB happiness OR AB enjoyment OR AB satisfaction OR AB ( resilience or resiliency or resilient ) OR AB mental ill-being OR AB mental illness OR AB mental ill-ness OR AB mental illbeing OR AB illbeing OR AB ill-being OR AB mental disorder OR AB psychological ill-being OR AB ( depression or depressive disorder* or depressive symptom* or major depressive disorder* ) OR AB ( anxiety or anxious or anxiety disorder* or anxiety symptom* ) OR AB ( stress or stressors or stress factor ) OR AB fatigue OR AB exhaustion OR AB burnout OR AB cognitive function OR AB cognitive functioning OR AB cognitive performance OR AB attention OR reaction time OR reaction OR exp cognition OR cognit* OR exp Memory OR Problem Solving OR attention OR cogniti* OR executive function OR executive functioning OR executive dysfunction OR memory OR problem*solving OR processing speed OR response inhibition OR inhibit* OR shifting OR switching OR neuropsychologic* OR cognitive control OR set-shifting OR cognitive flexibility OR impulse control OR mental flexibility OR mental set shifting OR self-control OR behavioural inhibition OR interference control OR reasoning OR accuracy OR AB psychosocial OR AB psycho*social OR AB confidence OR AB ( quality of life or QoL or QOL ) OR AB self-efficacy OR AB social support OR AB self-description OR AB ( self-esteem or self-image ) OR AB self-concept OR AB self-confidence OR AB ( self-competence or self-worth ) OR AB self-perception |
