# Supplementary material for: Physical activity and mental health in children and adolescents with intellectual disabilities: a meta-analysis using the RE-AIM framework
Source: Int J Behav Nutr Phys Act. 2022 Jul 7;19:80. doi: 10.1186/s12966-022-01312-1 (PMC9261031; doi:10.1186/s12966-022-01312-1)
Supplement: Supplementary file 3 — Additional file 3. Coding sheet for publications reporting on the RE-AIM framework components. [file 12966_2022_1312_MOESM3_ESM.docx]

**Additional file 3. Coding sheet for publications reporting on the RE-AIM framework components**

| **Impact Factor** |  | **Worksite** |  | |
| --- | --- | --- | --- | --- |
| **Author, Journal, Year, Page** |  | **PA types** |  | |
| **Reach** | **Reported (0 or 1)** | **Data** | **Comments** | |
| Method to identify target population |  |  |  | |
| Inclusion criteria |  |  |  | |
| Exclusion criteria |  |  |  | |
| Participation rate |  |  |  | |
| Representativeness |  |  |  | |
| Average across Reach Components |  |  |  | |
| **Effectiveness** | **Design** | **Conditions** | | |
| Design/conditions |  |  | | |
|  | **Reported (0 or 1)** | **Effect size and raw outcomes** | **P value** | **Comments** |
| Measures/results for at least one follow-up |  |  |  | |
| Intent to treat analysis method |  |  |  | |
| Quality-of-life or potential negative outcomes |  |  |  | |
| Percent attrition |  |  |  | |
| Average across Efficacy/Effectiveness Components |  |  |  | |
| **Adoption** | **Reported (0 or 1)** | **Data** | **Comments** | |
| Description of intervention location |  |  |  | |
| Description of staff who delivered intervention |  |  |  | |
| Method to identify staff who delivered intervention (target delivery agent) |  |  |  | |
| Level of expertise of delivery agent |  |  |  | |
| Inclusion/exclusion criteria of delivery agent or setting |  |  |  | |
| Adoption rate of delivery agent or setting |  |  |  | |
| Average across Adoption Components |  |  |  | |
| **Implementation** | **Report (0 or 1)** | **Data** | **Comments** | |
| Intervention duration and frequency |  |  |  | |
| Extent protocol delivered as intended (%) |  |  |  | |
| Measures of cost of implementation |  |  |  | |
| Average across Implementation Components |  |  |  | |
| **Maintenance** | **Reported (0 or 1)** | **Data** | **Comments** | |
| Assessed outcomes ≥6 months post intervention |  |  |  | |
| Indicators of program level maintenance |  |  |  | |
| Measures of cost of maintenance |  |  |  | |
| Average across Maintenance Components |  |  |  | |
