# Supplementary material for: Physical activity and mental health in children and adolescents with intellectual disabilities: a meta-analysis using the RE-AIM framework
Source: Int J Behav Nutr Phys Act. 2022 Jul 7;19:80. doi: 10.1186/s12966-022-01312-1 (PMC9261031; doi:10.1186/s12966-022-01312-1)
Supplement: Supplementary file 4 — Additional file 4. Proportion of PA interventions reporting the RE-AIM framework of included studies (n = 15). [file 12966_2022_1312_MOESM4_ESM.docx]

**Additional 4. Proportion of PA interventions reporting the RE-AIM framework of included studies (n = 15)**

|  | **Author & Year** | **Reach** | **Effectiveness** | **Adoption** | **Implementation** | **Maintenance** | **Total** |
| --- | --- | --- | --- | --- | --- | --- | --- |
| **A** | Choi & Cheung (2016) ^43^ | 40% | 75% | 16.7% | 33.3% | 0.0% | 33.0% |
|  | Maïano et al. (2002)^44^ | 100% | 25% | 16.7% | 33.3% | 33.3% | 41.7% |
|  | Maïano et al. (2001)^45^ | 60% | 50% | 0.0% | 33.3% | 33.3% | 35.3% |
|  | Ninot & Maïano (2007)^46^ | 80% | 25% | 0.0% | 33.3% | 0.0% | 27.7% |
|  | Ninot et al. (2005)^47^ | 80% | 25% | 0.0% | 33.3% | 0.0% | 27.7% |
|  | Ninot et al. (2000)^48^ | 80% | 25% | 0.0% | 33.3% | 0.0% | 27.7% |
|  | Özer et al. (2012)^49^ | 80% | 100% | 83.3% | 33.3% | 0.0% | 59.3% |
|  | Perić et al. (2021)^50^ | 80% | 100% | 50.0% | 66.7% | 33.3% | 66.0% |
|  | Ryuh et al. (2019)^51^ | 80% | 50% | 33.3% | 33.3% | 0.0% | 39.3% |
|  | *Average* | *75.6%* | *52.8%* | *22.2%* | *36.8%* | *11.0%* | *39.7%* |
| **B** | Chen et al. (2015)^52^ | 100% | 75% | 66.7% | 33.3% | 0.0% | 55.0% |
|  | Giagazoglou  et al. (2013)^53^ | 60% | 50% | 33.3% | 33.3% | 0.0% | 35.3% |
|  | Mazzoli et al. (2021)^54^ | 80% | 75% | 33.3% | 33.3% | 33.3% | 51.0% |
|  | Pise et al. (2018)^55^ | 60% | 75% | 16.7% | 33.3% | 0.0% | 37.0% |
|  | Vogt et al. (2013)^56^ | 80% | 50% | 0.0% | 33.3% | 0.0% | 32.7% |
|  | Yildirim et al. (2010)^57^ | 80% | 50% | 0.0% | 66.7% | 0.0% | 39.3% |
|  | *Average* | *76.7%* | *62.5%* | *25.0%* | *38.7%* | *5.5%* | *41.7%* |
| ***Total*** | | ***76%*** | ***56.7%*** | ***23.3%*** | ***37.8%*** | ***8.9%*** | ***40.5%*** |

*Note.* A: psychological health; B: cognitive function.
