# Supplementary material for: Physical activity and mental health in children and adolescents with intellectual disabilities: a meta-analysis using the RE-AIM framework
Source: Int J Behav Nutr Phys Act. 2022 Jul 7;19:80. doi: 10.1186/s12966-022-01312-1 (PMC9261031; doi:10.1186/s12966-022-01312-1)
Supplement: Supplementary file 5 — Additional file 5. Forest plot of subgroup analyses. [file 12966_2022_1312_MOESM5_ESM.docx]

**Additional file 5. Forest plot of subgroup analyses**

**Study location**

| **Study** | **Hedges’*g*** | **Standard error** | ***p* value** | **Weight** | **Hedges’*g* and 95% CI** |
| --- | --- | --- | --- | --- | --- |
| Choi & Cheung (2016) | 1.030 | 0.386 | 0.008 | 14.468 | 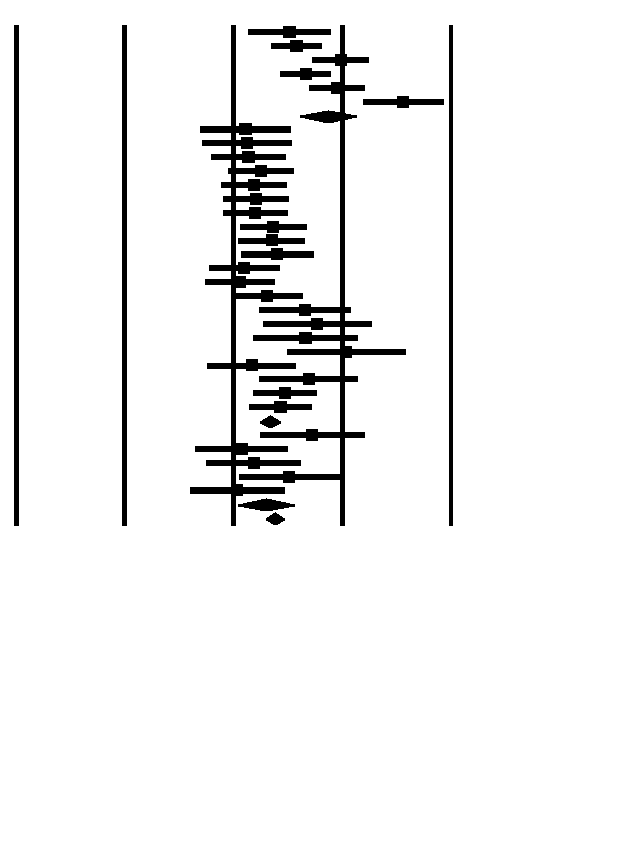 |
| Chen et al. (2015)–1 | 1.160 | 0.232 | 0.000 | 18.059 |  |
| Chen et al. (2015)–2 | 1.972 | 0.262 | 0.000 | 17.391 |  |
| Chen et al. (2015)–3 | 1.328 | 0.235 | 0.000 | 17.972 |  |
| Chen et al. (2015)–4 | 1.903 | 0.257 | 0.000 | 17.490 |  |
| Pise et al. (2018) | 3.126 | 0.380 | 0.000 | 14.620 |  |
| **Asia** | 1.730 | 0.264 | 0.000 | 100 |  |
| Maïano et al. (2002) | 0.222 | 0.419 | 0.596 | 3.771 |  |
| Maïano et al. (2002) | 0.251 | 0.420 | 0.550 | 3.767 |  |
| Maïano et al. (2001) | 0.276 | 0.346 | 0.426 | 4.968 |  |
| Ninot & Maïano (2007)–1 | 0.502 | 0.305 | 0.101 | 5.855 |  |
| Ninot & Maïano (2007)–2 | 0.372 | 0.304 | 0.221 | 5.901 |  |
| Ninot et al. (2005)–1 | 0.420 | 0.304 | 0.167 | 5.886 |  |
| Ninot et al. (2005)–2 | 0.401 | 0.304 | 0.187 | 5.892 |  |
| Ninot et al. (2000)–1 | 0.732 | 0.310 | 0.018 | 5.741 |  |
| Ninot et al. (2000)–2 | 0.702 | 0.310 | 0.023 | 5.758 |  |
| Özer et al. (2012)–1 | 0.810 | 0.338 | 0.017 | 5.137 |  |
| Özer et al. (2012)–2 | 0.203 | 0.326 | 0.534 | 5.393 |  |
| Özer et al. (2012)–3 | 0.116 | 0.325 | 0.721 | 5.405 |  |
| Özer et al. (2012)–4 | 0.622 | 0.333 | 0.062 | 5.246 |  |
| Perić et al. (2021) | 1.315 | 0.429 | 0.002 | 3.636 |  |
| Giagazoglou et al. (2013)–1 | 1.541 | 0.505 | 0.002 | 2.810 |  |
| Giagazoglou et al. (2013)–2 | 1.326 | 0.489 | 0.007 | 2.965 |  |
| Giagazoglou et al. (2013)–3 | 2.071 | 0.553 | 0.000 | 2.418 |  |
| Vogt et al. (2013)–1 | 0.333 | 0.413 | 0.421 | 3.855 |  |
| Vogt et al. (2013)–2 | 1.385 | 0.460 | 0.003 | 3.264 |  |
| Yildirim et al. (2010)–1 | 0.952 | 0.294 | 0.001 | 6.132 |  |
| Yildirim et al. (2010)–2 | 0.864 | 0.292 | 0.003 | 6.201 |  |
| **Europe** | 0.656 | 0.094 | 0.000 | 100 |  |
| Ryuh et al. (2019) | 1.448 | 0.486 | 0.003 | 18.294 |  |
| Mazzoli et al. (2021)–1 | 0.151 | 0.432 | 0.727 | 21.086 |  |
| Mazzoli et al. (2021)–2 | 0.372 | 0.441 | 0.400 | 20.581 |  |
| Mazzoli et al. (2021)–3 | 1.027 | 0.466 | 0.028 | 19.249 |  |
| Mazzoli et al. (2021)–4 | 0.072 | 0.437 | 0.870 | 20.790 |  |
| **America & Australia** | 0.586 | 0.261 | 0.025 | 100 |  |
| **Total** | 0.756 | 0.083 | 0.000 |  |  |
|  |  |  |  |  | **-4.00 -2.00 0.00 2.00 4.00** |

**Outcome provider**

| **Study** | **Hedges’*g*** | **Standard error** | ***p* value** | **Weight** | **Hedges’*g* and 95% CI** |
| --- | --- | --- | --- | --- | --- |
| Maïano et al. (2002) | 0.222 | 0.419 | 0.596 | 3.581 | 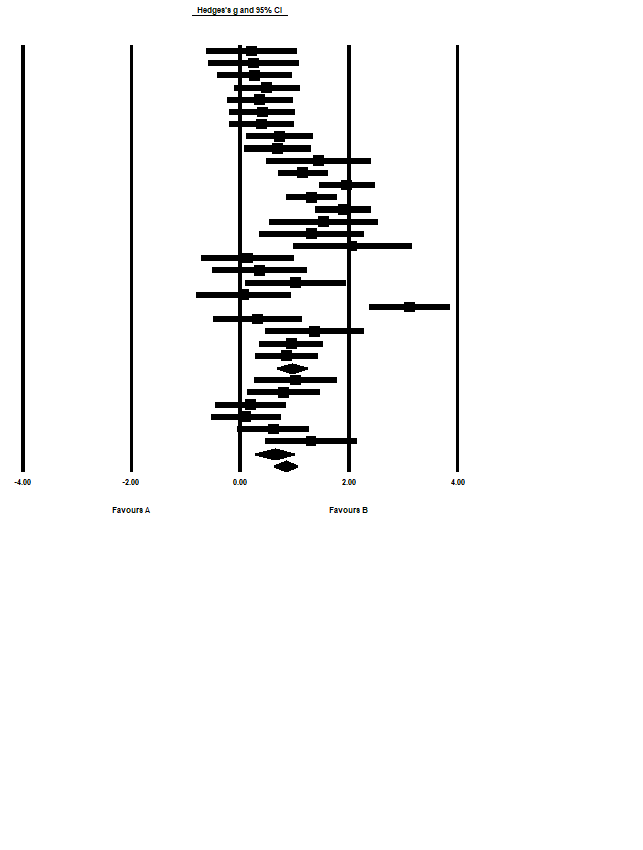 |
| Maïano et al. (2002) | 0.251 | 0.420 | 0.550 | 3.579 |  |
| Maïano et al. (2001) | 0.276 | 0.346 | 0.426 | 3.981 |  |
| Ninot & Maïano (2007)–1 | 0.502 | 0.305 | 0.101 | 4.204 |  |
| Ninot & Maïano (2007)–2 | 0.372 | 0.304 | 0.221 | 4.215 |  |
| Ninot et al. (2005)–1 | 0.420 | 0.304 | 0.167 | 4.211 |  |
| Ninot et al. (2005)–2 | 0.401 | 0.304 | 0.187 | 4.213 |  |
| Ninot et al. (2000)–1 | 0.732 | 0.310 | 0.018 | 4.178 |  |
| Ninot et al. (2000)–2 | 0.702 | 0.310 | 0.023 | 4.182 |  |
| Ryuh et al. (2019) | 1.448 | 0.486 | 0.003 | 3.232 |  |
| Chen et al. (2015)–1 | 1.160 | 0.232 | 0.000 | 4.589 |  |
| Chen et al. (2015)–2 | 1.972 | 0.262 | 0.000 | 4.438 |  |
| Chen et al. (2015)–3 | 1.328 | 0.235 | 0.000 | 4.570 |  |
| Chen et al. (2015)–4 | 1.903 | 0.257 | 0.000 | 4.460 |  |
| Giagazoglou et al. (2013)–1 | 1.541 | 0.505 | 0.002 | 3.134 |  |
| Giagazoglou et al. (2013)–2 | 1.326 | 0.489 | 0.007 | 3.216 |  |
| Giagazoglou et al. (2013)–3 | 2.071 | 0.553 | 0.000 | 2.904 |  |
| Mazzoli et al. (2021)–1 | 0.151 | 0.432 | 0.727 | 3.513 |  |
| Mazzoli et al. (2021)–2 | 0.372 | 0.441 | 0.400 | 3.464 |  |
| Mazzoli et al. (2021)–3 | 1.027 | 0.466 | 0.028 | 3.332 |  |
| Mazzoli et al. (2021)–4 | 0.072 | 0.437 | 0.870 | 3.484 |  |
| Pise et al. (2018) | 3.126 | 0.380 | 0.000 | 3.797 |  |
| Vogt et al. (2013)–1 | 0.333 | 0.413 | 0.421 | 3.614 |  |
| Vogt et al. (2013)–2 | 1.385 | 0.460 | 0.003 | 3.363 |  |
| Yildirim et al. (2010)–1 | 0.952 | 0.294 | 0.001 | 4.265 |  |
| Yildirim et al. (2010)–2 | 0.864 | 0.292 | 0.003 | 4.280 |  |
| **Self-report** | 0.953 | 0.141 | 0.000 | 100 |  |
| Choi & Cheung (2016) | 1.030 | 0.386 | 0.008 | 14.865 |  |
| Özer et al. (2012)–1 | 0.810 | 0.338 | 0.017 | 17.588 |  |
| Özer et al. (2012)–2 | 0.203 | 0.326 | 0.534 | 18.366 |  |
| Özer et al. (2012)–3 | 0.116 | 0.325 | 0.721 | 18.403 |  |
| Özer et al. (2012)–4 | 0.622 | 0.333 | 0.062 | 17.921 |  |
| Perić et al. (2021) | 1.315 | 0.429 | 0.002 | 12.857 |  |
| **Teacher or parent**  **proxy-report** | 0.634 | 0.183 | 0.001 | 100 |  |
| **Total** | 0.835 | 0.112 | 0.000 |  |  |
|  |  |  |  |  | **-4.00 -2.00 0.00 2.00 4.00** |

**Study design**

| **Study** | **Hedges’*g*** | **Standard error** | ***p* value** | **Weight** | **Hedges’*g* and 95% CI** |
| --- | --- | --- | --- | --- | --- |
| Maïano et al. (2002) | 0.222 | 0.419 | 0.596 | 6.306 |  |
| Maïano et al. (2002) | 0.251 | 0.420 | 0.550 | 6.295 |  |
| Ninot & Maïano (2007)–1 | 0.502 | 0.305 | 0.101 | 11.880 |  |
| Ninot & Maïano (2007)–2 | 0.372 | 0.304 | 0.221 | 12.032 |  |
| Ninot et al. (2005)–1 | 0.420 | 0.304 | 0.167 | 11.980 |  |
| Ninot et al. (2005)–2 | 0.401 | 0.304 | 0.187 | 12.001 |  |
| Ninot et al. (2000)–1 | 0.732 | 0.310 | 0.018 | 11.515 |  |
| Ninot et al. (2000)–2 | 0.702 | 0.310 | 0.023 | 11.568 |  |
| Ryuh et al. (2019) | 1.448 | 0.486 | 0.003 | 4.700 |  |
| Vogt et al. (2013)–1 | 0.333 | 0.413 | 0.421 | 6.491 |  |
| Vogt et al. (2013)–2 | 1.385 | 0.460 | 0.003 | 5.233 |  |
| **Non-RCT** | 0.560 | 0.105 | 0.000 | 100 | 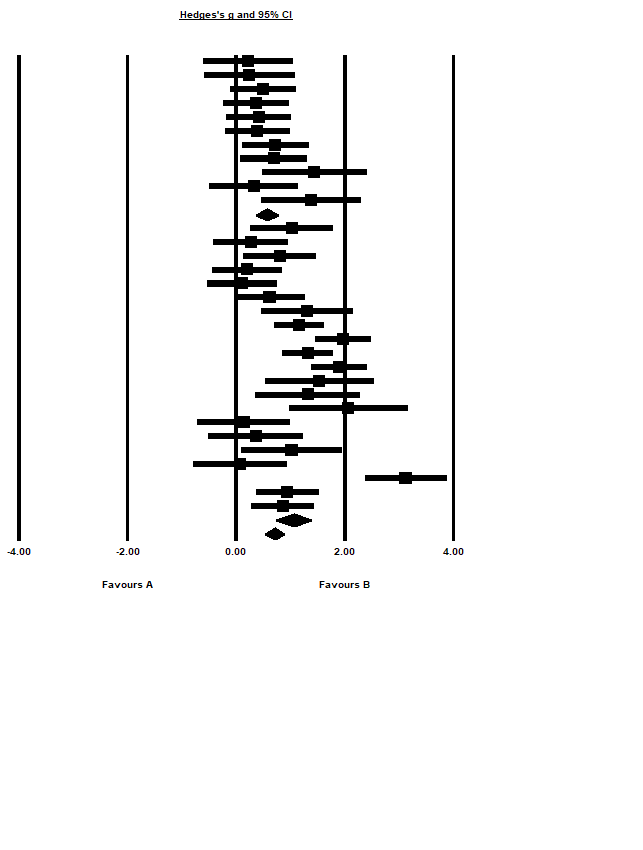 |
| Choi & Cheung (2016) | 1.030 | 0.386 | 0.008 | 4.658 |  |
| Maïano et al. (2001) | 0.276 | 0.346 | 0.426 | 4.908 |  |
| Özer et al. (2012)–1 | 0.810 | 0.338 | 0.017 | 4.961 |  |
| Özer et al. (2012)–2 | 0.203 | 0.326 | 0.534 | 5.037 |  |
| Özer et al. (2012)–3 | 0.116 | 0.325 | 0.721 | 5.040 |  |
| Özer et al. (2012)–4 | 0.622 | 0.333 | 0.062 | 4.994 |  |
| Perić et al. (2021) | 1.315 | 0.429 | 0.002 | 4.388 |  |
| Chen et al. (2015)–1 | 1.160 | 0.232 | 0.000 | 5.591 |  |
| Chen et al. (2015)–2 | 1.972 | 0.262 | 0.000 | 5.423 |  |
| Chen et al. (2015)–3 | 1.328 | 0.235 | 0.000 | 5.569 |  |
| Chen et al. (2015)–4 | 1.903 | 0.257 | 0.000 | 5.448 |  |
| Giagazoglou et al. (2013)–1 | 1.541 | 0.505 | 0.002 | 3.931 |  |
| Giagazoglou et al. (2013)–2 | 1.326 | 0.489 | 0.007 | 4.028 |  |
| Giagazoglou et al. (2013)–3 | 2.071 | 0.553 | 0.000 | 3.660 |  |
| Mazzoli et al. (2021)–1 | 0.151 | 0.432 | 0.727 | 4.372 |  |
| Mazzoli et al. (2021)–2 | 0.372 | 0.441 | 0.400 | 4.316 |  |
| Mazzoli et al. (2021)–3 | 1.027 | 0.466 | 0.028 | 4.162 |  |
| Mazzoli et al. (2021)–4 | 0.072 | 0.437 | 0.870 | 4.340 |  |
| Pise et al. (2018) | 3.126 | 0.380 | 0.000 | 4.699 |  |
| Yildirim et al. (2010)–1 | 0.952 | 0.294 | 0.001 | 5.230 |  |
| Yildirim et al. (2010)–2 | 0.864 | 0.292 | 0.003 | 5.246 |  |
| **RCT** | 1.056 | 0.163 | 0.000 | 100 |  |
| **Total** | 0.706 | 0.089 | 0.000 |  |  |
|  |  |  |  |  | **-4.00 -2.00 0.00 2.00 4.00** |

*Note.* RCT: randomized control trial.

**Age**

| **Study** | **Hedges’*g*** | **Standard error** | ***p* value** | **Weight** | **Hedges’*g* and 95% CI** |
| --- | --- | --- | --- | --- | --- |
| Giagazoglou et al. (2013)–1 | 1.541 | 0.505 | 0.002 | 2.810 | 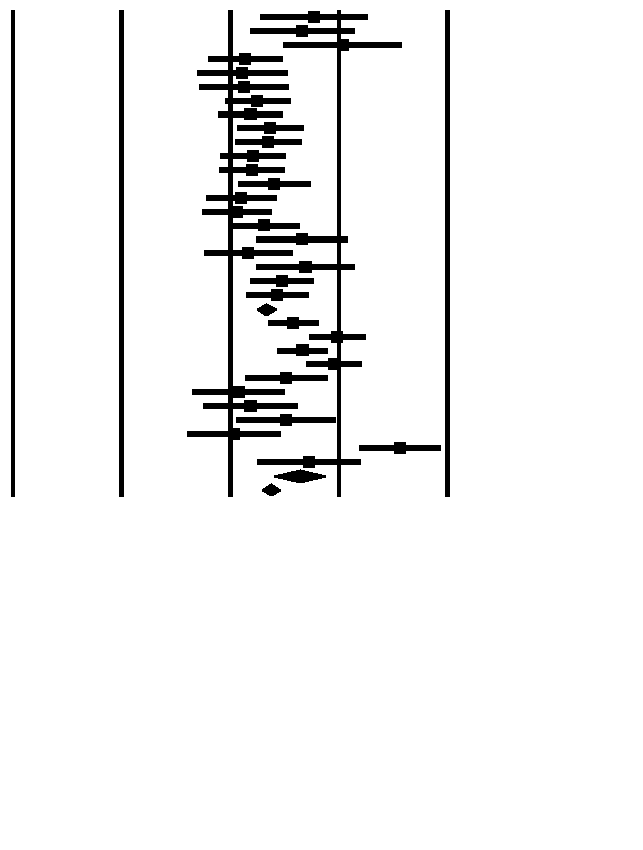 |
| Giagazoglou et al. (2013)–2 | 1.326 | 0.489 | 0.007 | 2.965 |  |
| Giagazoglou et al. (2013)–3 | 2.071 | 0.553 | 0.000 | 2.418 |  |
| Maïano et al. (2001) | 0.276 | 0.346 | 0.426 | 4.968 |  |
| Maïano et al. (2002) | 0.222 | 0.419 | 0.596 | 3.771 |  |
| Maïano et al. (2002) | 0.251 | 0.420 | 0.550 | 3.767 |  |
| Ninot & Maïano (2007)–1 | 0.502 | 0.305 | 0.101 | 5.855 |  |
| Ninot & Maïano (2007)–2 | 0.372 | 0.304 | 0.221 | 5.901 |  |
| Ninot et al. (2005)–1 | 0.732 | 0.310 | 0.018 | 5.741 |  |
| Ninot et al. (2005)–2 | 0.702 | 0.310 | 0.023 | 5.758 |  |
| Ninot et al. (2000)–1 | 0.420 | 0.304 | 0.167 | 5.886 |  |
| Ninot et al. (2000)–2 | 0.401 | 0.304 | 0.187 | 5.892 |  |
| Özer et al. (2012)–1 | 0.810 | 0.338 | 0.017 | 5.137 |  |
| Özer et al. (2012)–2 | 0.203 | 0.326 | 0.534 | 5.393 |  |
| Özer et al. (2012)–3 | 0.116 | 0.325 | 0.721 | 5.405 |  |
| Özer et al. (2012)–4 | 0.622 | 0.333 | 0.062 | 5.246 |  |
| Perić et al. (2021) | 1.315 | 0.429 | 0.002 | 3.636 |  |
| Vogt et al. (2013)–1 | 0.333 | 0.413 | 0.421 | 3.855 |  |
| Vogt et al. (2013)–2 | 1.385 | 0.460 | 0.003 | 3.264 |  |
| Yildirim et al. (2010)–1 | 0.952 | 0.294 | 0.001 | 6.132 |  |
| Yildirim et al. (2010)–2 | 0.864 | 0.292 | 0.003 | 6.201 |  |
| **Adolescents** | 0.656 | 0.094 | 0.000 | 100 |  |
| Chen et al. (2015)–1 | 1.160 | 0.232 | 0.000 | 10.452 |  |
| Chen et al. (2015)–2 | 1.972 | 0.262 | 0.000 | 10.175 |  |
| Chen et al. (2015)–3 | 1.328 | 0.235 | 0.000 | 10.416 |  |
| Chen et al. (2015)–4 | 1.903 | 0.257 | 0.000 | 10.216 |  |
| Choi & Cheung (2016) | 1.030 | 0.386 | 0.008 | 8.889 |  |
| Mazzoli et al. (2021)–1 | 0.151 | 0.432 | 0.727 | 8.397 |  |
| Mazzoli et al. (2021)–2 | 0.372 | 0.441 | 0.400 | 8.299 |  |
| Mazzoli et al. (2021)–3 | 1.027 | 0.466 | 0.028 | 8.031 |  |
| Mazzoli et al. (2021)–4 | 0.072 | 0.437 | 0.870 | 8.340 |  |
| Pise et al. (2018) | 3.126 | 0.380 | 0.000 | 8.959 |  |
| Ryuh et al. (2019) | 1.448 | 0.486 | 0.003 | 7.827 |  |
| **Children** | 1.272 | 0.238 | 0.000 | 100 |  |
| **Total** | 0.738 | 0.087 | 0.000 |  |  |
|  |  |  |  |  | **-4.00 -2.00 0.00 2.00 4.00** |

**Sex**

| **Study** | **Hedges’*g*** | **Standard error** | ***p* value** | **Weight** | **Hedges’*g* and 95% CI** |
| --- | --- | --- | --- | --- | --- |
| Giagazoglou et al. (2013)–1 | 1.541 | 0.505 | 0.002 | 6.687 |  |
| Giagazoglou et al. (2013)–2 | 1.326 | 0.489 | 0.007 | 6.938 |  |
| Giagazoglou et al. (2013)–3 | 2.071 | 0.553 | 0.000 | 6.013 |  |
| Maïano et al. (2001) | 0.276 | 0.346 | 0.426 | 9.567 |  |
| Maïano et al. (2002) | 0.222 | 0.419 | 0.596 | 8.122 |  |
| Maïano et al. (2002) | 0.251 | 0.420 | 0.550 | 8.116 |  |
| Özer et al. (2012)–1 | 0.810 | 0.338 | 0.017 | 9.746 | 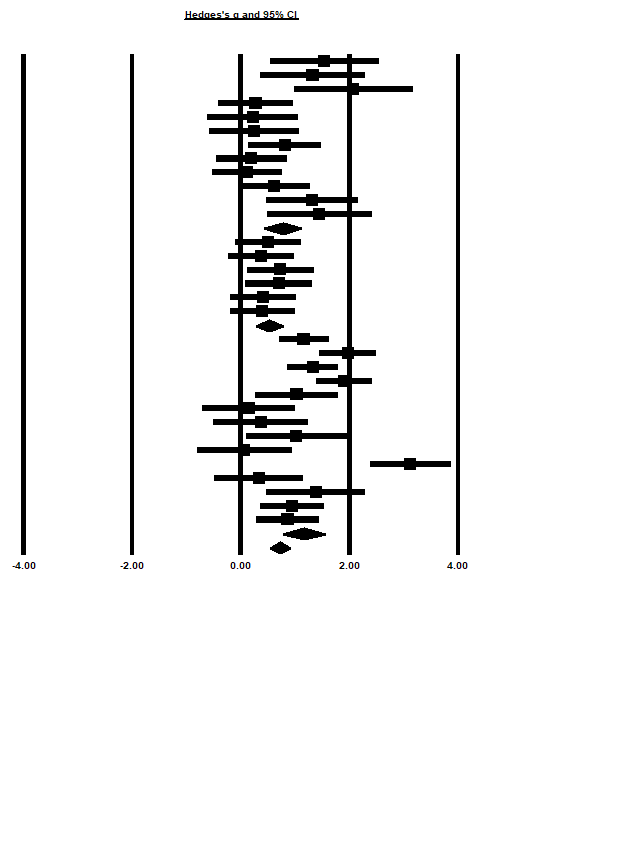 |
| Özer et al. (2012)–2 | 0.203 | 0.326 | 0.534 | 10.009 |  |
| Özer et al. (2012)–3 | 0.116 | 0.325 | 0.721 | 10.021 |  |
| Özer et al. (2012)–4 | 0.622 | 0.333 | 0.062 | 9.859 |  |
| Perić et al. (2021) | 1.315 | 0.429 | 0.002 | 7.937 |  |
| Ryuh et al. (2019) | 1.448 | 0.486 | 0.003 | 6.987 |  |
| **Boy only** | 0.762 | 0.173 | 0.000 | 100 |  |
| Ninot & Maïano (2007)–1 | 0.502 | 0.305 | 0.101 | 16.738 |  |
| Ninot & Maïano (2007)–2 | 0.372 | 0.304 | 0.221 | 16.952 |  |
| Ninot et al. (2005)–1 | 0.732 | 0.310 | 0.018 | 16.224 |  |
| Ninot et al. (2005)–2 | 0.702 | 0.310 | 0.023 | 16.298 |  |
| Ninot et al. (2000)–1 | 0.420 | 0.304 | 0.167 | 16.879 |  |
| Ninot et al. (2000)–2 | 0.401 | 0.304 | 0.187 | 16.909 |  |
| **Girl only** | 0.519 | 0.125 | 0.000 | 100 |  |
| Chen et al. (2015)–1 | 1.160 | 0.232 | 0.000 | 8.252 |  |
| Chen et al. (2015)–2 | 1.972 | 0.262 | 0.000 | 8.004 |  |
| Chen et al. (2015)–3 | 1.328 | 0.235 | 0.000 | 8.220 |  |
| Chen et al. (2015)–4 | 1.903 | 0.257 | 0.000 | 8.041 |  |
| Choi & Cheung (2016) | 1.030 | 0.386 | 0.008 | 6.877 |  |
| Mazzoli et al. (2021)–1 | 0.151 | 0.432 | 0.727 | 6.455 |  |
| Mazzoli et al. (2021)–2 | 0.372 | 0.441 | 0.400 | 6.372 |  |
| Mazzoli et al. (2021)–3 | 1.027 | 0.466 | 0.028 | 6.145 |  |
| Mazzoli et al. (2021)–4 | 0.072 | 0.437 | 0.870 | 6.407 |  |
| Pise et al. (2018) | 3.126 | 0.380 | 0.000 | 6.937 |  |
| Vogt et al. (2013)–1 | 0.333 | 0.413 | 0.421 | 6.627 |  |
| Vogt et al. (2013)–2 | 1.385 | 0.460 | 0.003 | 6.199 |  |
| Yildirim et al. (2010)–1 | 0.952 | 0.294 | 0.001 | 7.720 |  |
| Yildirim et al. (2010)–2 | 0.864 | 0.292 | 0.003 | 7.744 |  |
| **Mixed** | 1.153 | 0.199 | 0.000 | 100 |  |
| **Total** | 0.716 | 0.090 | 0.000 |  |  |
|  |  |  |  |  | **-4.00 -2.00 0.00 2.00 4.00** |

**Intellectual disability level**

| **Study** | **Hedges’*g*** | **Standard error** | ***p* value** | **Weight** | **Hedges’*g* and 95% CI** |
| --- | --- | --- | --- | --- | --- |
| Choi & Cheung (2016) | 1.030 | 0.386 | 0.008 | 9.512 |  |
| Maïano et al. (2002) | 0.222 | 0.419 | 0.596 | 8.879 |  |
| Maïano et al. (2002) | 0.251 | 0.420 | 0.550 | 8.872 |  |
| Chen et al. (2015)–1 | 1.160 | 0.232 | 0.000 | 12.777 |  |
| Chen et al. (2015)–2 | 1.972 | 0.262 | 0.000 | 12.132 |  |
| Chen et al. (2015)–3 | 1.328 | 0.235 | 0.000 | 12.693 |  |
| Chen et al. (2015)–4 | 1.903 | 0.257 | 0.000 | 12.227 |  |
| Yildirim et al. (2010)–1 | 0.952 | 0.294 | 0.001 | 11.424 |  |
| Yildirim et al. (2010)–2 | 0.864 | 0.292 | 0.003 | 11.483 |  |
| **Mild ID** | 1.137 | 0.189 | 0.000 | 100 |  |
| Maïano et al. (2001) | 0.276 | 0.346 | 0.426 | 6.647 |  |
| Ninot & Maïano (2007)–1 | 0.502 | 0.305 | 0.101 | 6.980 |  |
| Ninot & Maïano (2007)–2 | 0.372 | 0.304 | 0.221 | 6.995 |  |
| Ninot et al. (2005)–1 | 0.420 | 0.304 | 0.167 | 6.990 |  |
| Ninot et al. (2005)–2 | 0.401 | 0.304 | 0.187 | 6.992 |  |
| Ninot et al. (2000)–1 | 0.732 | 0.310 | 0.018 | 6.941 |  |
| Ninot et al. (2000)–2 | 0.702 | 0.310 | 0.023 | 6.947 | 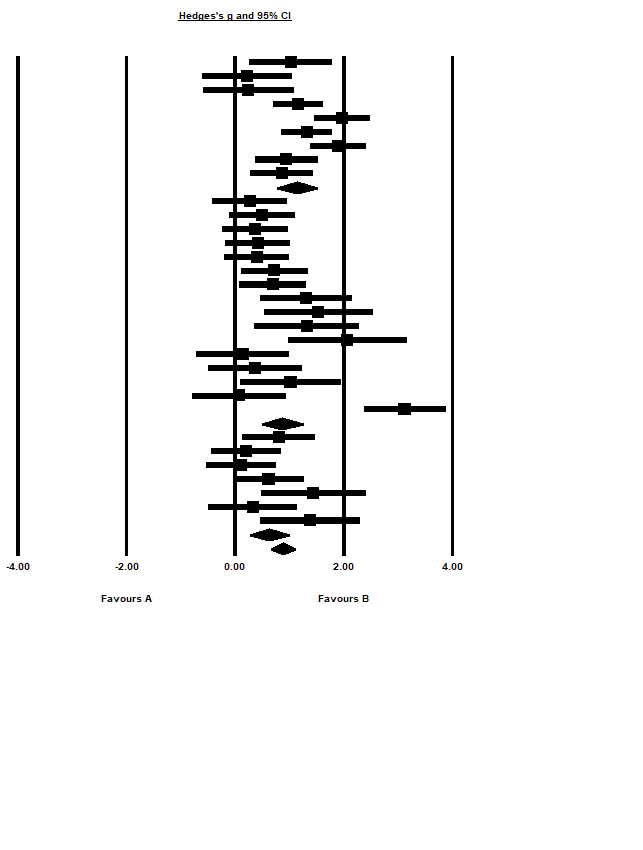 |
| Perić et al. (2021) | 1.315 | 0.429 | 0.002 | 5.959 |  |
| Giagazoglou et al. (2013)–1 | 1.541 | 0.505 | 0.002 | 5.352 |  |
| Giagazoglou et al. (2013)–2 | 1.326 | 0.489 | 0.007 | 5.480 |  |
| Giagazoglou et al. (2013)–3 | 2.071 | 0.553 | 0.000 | 4.989 |  |
| Mazzoli et al. (2021)–1 | 0.151 | 0.432 | 0.727 | 5.938 |  |
| Mazzoli et al. (2021)–2 | 0.372 | 0.441 | 0.400 | 5.864 |  |
| Mazzoli et al. (2021)–3 | 1.027 | 0.466 | 0.028 | 5.659 |  |
| Mazzoli et al. (2021)–4 | 0.072 | 0.437 | 0.870 | 5.895 |  |
| Pise et al. (2018) | 3.126 | 0.380 | 0.000 | 6.371 |  |
| **Mild to moderate ID** | 0.865 | 0.193 | 0.000 | 100 |  |
| Özer et al. (2012)–1 | 0.810 | 0.338 | 0.017 | 16.080 |  |
| Özer et al. (2012)–2 | 0.203 | 0.326 | 0.534 | 16.703 |  |
| Özer et al. (2012)–3 | 0.116 | 0.325 | 0.721 | 16.732 |  |
| Özer et al. (2012)–4 | 0.622 | 0.333 | 0.062 | 16.347 |  |
| Ryuh et al. (2019) | 1.448 | 0.486 | 0.003 | 10.299 |  |
| Vogt et al. (2013)–1 | 0.333 | 0.413 | 0.421 | 12.752 |  |
| Vogt et al. (2013)–2 | 1.385 | 0.460 | 0.003 | 11.086 |  |
| **Overall ID** | 0.630 | 0.187 | 0.001 | 100 |  |
| **Total** | 0.876 | 0.109 | 0.000 |  |  |
|  |  |  |  |  | **-4.00 -2.00 0.00 2.00 4.00** |

**Intervention type**

| **Study** | **Hedges’*g*** | **Standard error** | ***p* value** | **Weight** | **Hedges’*g* and 95% CI** |
| --- | --- | --- | --- | --- | --- |
| Chen et al. (2015)–1 | 1.160 | 0.232 | 0.000 | 29.233 | 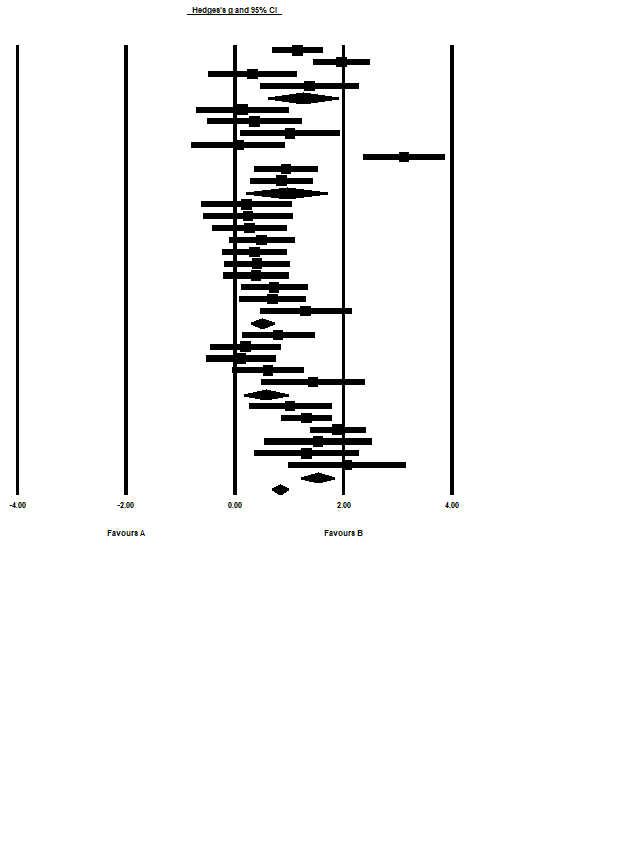 |
| Chen et al. (2015)–2 | 1.972 | 0.262 | 0.000 | 28.107 |  |
| Vogt et al. (2013)–1 | 0.333 | 0.413 | 0.421 | 22.194 |  |
| Vogt et al. (2013)–2 | 1.385 | 0.460 | 0.003 | 20.466 |  |
| **Aerobic exercise** | 1.251 | 0.329 | 0.000 | 100 |  |
| Mazzoli et al. (2021)–1 | 0.151 | 0.432 | 0.727 | 13.857 |  |
| Mazzoli et al. (2021)–2 | 0.372 | 0.441 | 0.400 | 13.752 |  |
| Mazzoli et al. (2021)–3 | 1.027 | 0.466 | 0.028 | 13.458 |  |
| Mazzoli et al. (2021)–4 | 0.072 | 0.437 | 0.870 | 13.796 |  |
| Pise et al. (2018) | 3.126 | 0.380 | 0.000 | 14.447 |  |
| Yildirim et al. (2010)–1 | 0.952 | 0.294 | 0.001 | 15.333 |  |
| Yildirim et al. (2010)–2 | 0.864 | 0.292 | 0.003 | 15.359 |  |
| **Cognitive exercise** | 0.950 | 0.380 | 0.012 | 100 |  |
| Maïano et al. (2002) | 0.222 | 0.419 | 0.596 | 6.380 |  |
| Maïano et al. (2002) | 0.251 | 0.420 | 0.550 | 6.370 |  |
| Maïano et al. (2001) | 0.276 | 0.346 | 0.426 | 9.351 |  |
| Ninot & Maïano (2007)–1 | 0.502 | 0.305 | 0.101 | 12.020 |  |
| Ninot & Maïano (2007)–2 | 0.372 | 0.304 | 0.221 | 12.175 |  |
| Ninot et al. (2005)–1 | 0.420 | 0.304 | 0.167 | 12.122 |  |
| Ninot et al. (2005)–2 | 0.401 | 0.304 | 0.187 | 12.143 |  |
| Ninot et al. (2000)–1 | 0.732 | 0.310 | 0.018 | 11.651 |  |
| Ninot et al. (2000)–2 | 0.702 | 0.310 | 0.023 | 11.705 |  |
| Perić et al. (2021) | 1.315 | 0.429 | 0.002 | 6.083 |  |
| **Competitive sports** | 0.509 | 0.106 | 0.000 | 100 |  |
| Özer et al. (2012)–1 | 0.810 | 0.338 | 0.017 | 21.142 |  |
| Özer et al. (2012)–2 | 0.203 | 0.326 | 0.534 | 22.005 |  |
| Özer et al. (2012)–3 | 0.116 | 0.325 | 0.721 | 22.046 |  |
| Özer et al. (2012)–4 | 0.622 | 0.333 | 0.062 | 21.512 |  |
| Ryuh et al. (2019) | 1.448 | 0.486 | 0.003 | 13.295 |  |
| **Non-competitive sports** | 0.568 | 0.209 | 0.007 | 100 |  |
| Choi & Cheung (2016) | 1.030 | 0.386 | 0.008 | 14.134 |  |
| Chen et al. (2015)–3 | 1.328 | 0.235 | 0.000 | 32.491 |  |
| Chen et al. (2015)–4 | 1.903 | 0.257 | 0.000 | 28.304 |  |
| Giagazoglou et al. (2013)–1 | 1.541 | 0.505 | 0.002 | 8.624 |  |
| Giagazoglou et al. (2013)–2 | 1.326 | 0.489 | 0.007 | 9.171 |  |
| Giagazoglou et al. (2013)–3 | 2.071 | 0.553 | 0.000 | 7.275 |  |
| **Therapeutic exercise** | 1.521 | 0.153 | 0.000 | 100 |  |
| **Total** | 0.827 | 0.076 | 0.000 |  |  |
|  |  |  |  |  | **-4.00 -2.00 0.00 2.00 4.00** |

**Intervention setting**

| **Study** | **Hedges’*g*** | **Standard error** | ***p* value** | **Weight** | **Hedges’*g* and 95% CI** |
| --- | --- | --- | --- | --- | --- |
| Maïano et al. (2002) | 0.222 | 0.419 | 0.596 | 4.653 |  |
| Maïano et al. (2002) | 0.251 | 0.420 | 0.550 | 4.650 |  |
| Maïano et al. (2001) | 0.276 | 0.346 | 0.426 | 5.306 |  |
| Ninot & Maïano (2007)–1 | 0.502 | 0.305 | 0.101 | 5.686 |  |
| Ninot & Maïano (2007)–2 | 0.372 | 0.304 | 0.221 | 5.704 |  |
| Ninot et al. (2005)–1 | 0.420 | 0.304 | 0.167 | 5.698 |  |
| Ninot et al. (2005)–2 | 0.401 | 0.304 | 0.187 | 5.701 |  |
| Ninot et al. (2000)–1 | 0.732 | 0.310 | 0.018 | 5.641 | 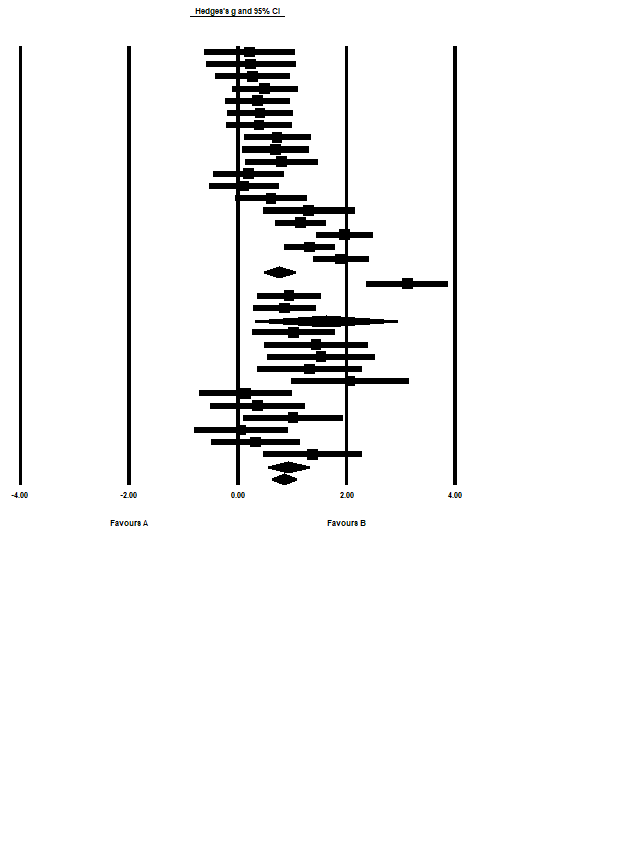 |
| Ninot et al. (2000)–2 | 0.702 | 0.310 | 0.023 | 5.648 |  |
| Özer et al. (2012)–1 | 0.810 | 0.338 | 0.017 | 5.384 |  |
| Özer et al. (2012)–2 | 0.203 | 0.326 | 0.534 | 5.497 |  |
| Özer et al. (2012)–3 | 0.116 | 0.325 | 0.721 | 5.502 |  |
| Özer et al. (2012)–4 | 0.622 | 0.333 | 0.062 | 5.433 |  |
| Perić et al. (2021) | 1.315 | 0.429 | 0.002 | 4.566 |  |
| Chen et al. (2015)–1 | 1.160 | 0.232 | 0.000 | 6.367 |  |
| Chen et al. (2015)–2 | 1.972 | 0.262 | 0.000 | 6.095 |  |
| Chen et al. (2015)–3 | 1.328 | 0.235 | 0.000 | 6.332 |  |
| Chen et al. (2015)–4 | 1.903 | 0.257 | 0.000 | 6.135 |  |
| **Community** | 0.764 | 0.145 | 0.000 | 100 |  |
| Pise et al. (2018) | 3.126 | 0.380 | 0.000 | 32.381 |  |
| Yildirim et al. (2010)–1 | 0.952 | 0.294 | 0.001 | 33.789 |  |
| Yildirim et al. (2010)–2 | 0.864 | 0.292 | 0.003 | 33.830 |  |
| **Not reported** | 1.626 | 0.669 | 0.015 | 100 |  |
| Choi & Cheung (2016) | 1.030 | 0.386 | 0.008 | 10.710 |  |
| Ryuh et al. (2019) | 1.448 | 0.486 | 0.003 | 8.548 |  |
| Giagazoglou et al. (2013)–1 | 1.541 | 0.505 | 0.002 | 8.182 |  |
| Giagazoglou et al. (2013)–2 | 1.326 | 0.489 | 0.007 | 8.489 |  |
| Giagazoglou et al. (2013)–3 | 2.071 | 0.553 | 0.000 | 7.356 |  |
| Mazzoli et al. (2021)–1 | 0.151 | 0.432 | 0.727 | 9.655 |  |
| Mazzoli et al. (2021)–2 | 0.372 | 0.441 | 0.400 | 9.458 |  |
| Mazzoli et al. (2021)–3 | 1.027 | 0.466 | 0.028 | 8.932 |  |
| Mazzoli et al. (2021)–4 | 0.072 | 0.437 | 0.870 | 9.540 |  |
| Vogt et al. (2013)–1 | 0.333 | 0.413 | 0.421 | 10.075 |  |
| Vogt et al. (2013)–2 | 1.385 | 0.460 | 0.003 | 9.055 |  |
| **School** | 0.932 | 0.192 | 0.000 | 100 |  |
| **Total** | 0.849 | 0.114 | 0.000 |  |  |
|  |  |  |  |  | **-4.00 -2.00 0.00 2.00 4.00** |

**Intervention duration per week**

| **Study** | **Hedges’*g*** | **Standard error** | ***p* value** | **Weight** | **Hedges’*g* and 95% CI** |
| --- | --- | --- | --- | --- | --- |
| Maïano et al. (2002) | 0.222 | 0.419 | 0.596 | 4.397 |  |
| Maïano et al. (2002) | 0.251 | 0.420 | 0.550 | 4.391 |  |
| Maïano et al. (2001) | 0.276 | 0.346 | 0.426 | 5.810 |  |
| Ninot & Maïano (2007)–1 | 0.502 | 0.305 | 0.101 | 6.863 |  |
| Ninot & Maïano (2007)–2 | 0.372 | 0.304 | 0.221 | 6.918 |  |
| Ninot et al. (2005)–1 | 0.420 | 0.304 | 0.167 | 6.900 |  |
| Ninot et al. (2005)–2 | 0.401 | 0.304 | 0.187 | 6.907 | 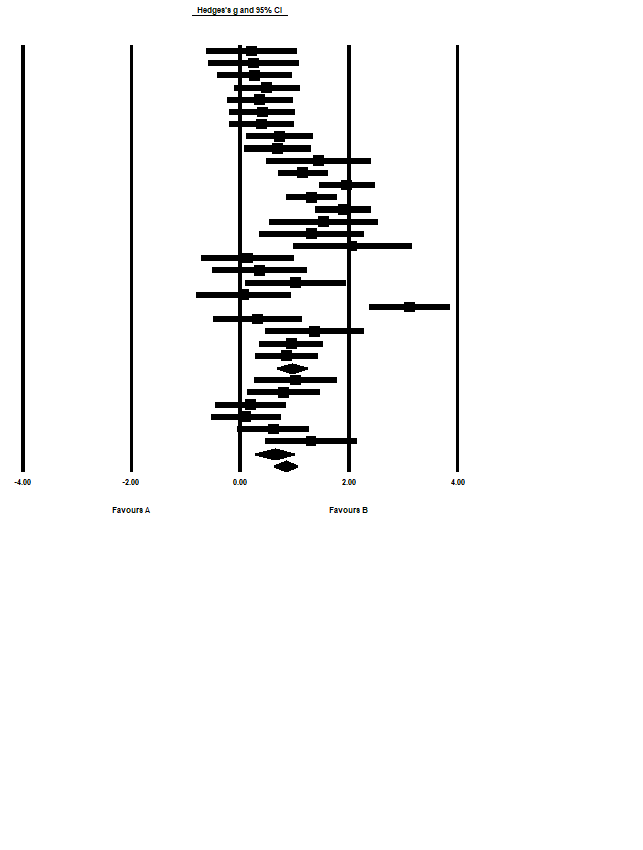 |
| Ninot et al. (2000)–1 | 0.732 | 0.310 | 0.018 | 6.728 |  |
| Ninot et al. (2000)–2 | 0.702 | 0.310 | 0.023 | 6.747 |  |
| Perić et al. (2021) | 1.315 | 0.429 | 0.002 | 4.238 |  |
| Giagazoglou et al. (2013)–1 | 1.541 | 0.505 | 0.002 | 3.268 |  |
| Giagazoglou et al. (2013)–2 | 1.326 | 0.489 | 0.007 | 3.449 |  |
| Giagazoglou et al. (2013)–3 | 2.071 | 0.553 | 0.000 | 2.809 |  |
| Mazzoli et al. (2021)–1 | 0.151 | 0.432 | 0.727 | 4.199 |  |
| Mazzoli et al. (2021)–2 | 0.372 | 0.441 | 0.400 | 4.065 |  |
| Mazzoli et al. (2021)–3 | 1.027 | 0.466 | 0.028 | 3.722 |  |
| Mazzoli et al. (2021)–4 | 0.072 | 0.437 | 0.870 | 4.120 |  |
| Yildirim et al. (2010)–1 | 0.952 | 0.294 | 0.001 | 7.194 |  |
| Yildirim et al. (2010)–2 | 0.864 | 0.292 | 0.003 | 7.276 |  |
| **≤120 min/week** | 0.654 | 0.100 | 0.000 | 100 |  |
| Choi & Cheung (2016) | 1.030 | 0.386 | 0.008 | 8.513 |  |
| Özer et al. (2012)–1 | 0.810 | 0.338 | 0.017 | 8.977 |  |
| Özer et al. (2012)–2 | 0.203 | 0.326 | 0.534 | 9.092 |  |
| Özer et al. (2012)–3 | 0.116 | 0.325 | 0.721 | 9.097 |  |
| Özer et al. (2012)–4 | 0.622 | 0.333 | 0.062 | 9.027 |  |
| Ryuh et al. (2019) | 1.448 | 0.486 | 0.003 | 7.546 |  |
| Chen et al. (2015)–1 | 1.160 | 0.232 | 0.000 | 9.914 |  |
| Chen et al. (2015)–2 | 1.972 | 0.262 | 0.000 | 9.668 |  |
| Chen et al. (2015)–3 | 1.328 | 0.235 | 0.000 | 9.883 |  |
| Chen et al. (2015)–4 | 1.903 | 0.257 | 0.000 | 9.705 |  |
| Pise et al. (2018) | 3.126 | 0.380 | 0.000 | 8.577 |  |
| **>120 min/week** | 1.244 | 0.240 | 0.000 | 100 |  |
| Vogt et al. (2013)–1 | 0.333 | 0.413 | 0.421 | 51.856 |  |
| Vogt et al. (2013)–2 | 1.385 | 0.460 | 0.003 | 48.144 |  |
| **Not reported** | 0.839 | 0.526 | 0.110 |  |  |
| **Total** | 0.745 | 0.091 | 0.000 |  |  |
|  |  |  |  |  | **-4.00 -2.00 0.00 2.00 4.00** |
